# Well-Defined Nanostructures by Block Copolymers and Mass Transport Applications in Energy Conversion

**DOI:** 10.3390/polym14214568

**Published:** 2022-10-28

**Authors:** Shuhui Ma, Yushuang Hou, Jinlin Hao, Cuncai Lin, Jiawei Zhao, Xin Sui

**Affiliations:** College of Materials Science and Engineering, Qingdao University, Qingdao 266071, China

**Keywords:** block copolymer, self-assembly, nanochannels, membranes, energy conversion

## Abstract

With the speedy progress in the research of nanomaterials, self-assembly technology has captured the high-profile interest of researchers because of its simplicity and ease of spontaneous formation of a stable ordered aggregation system. The self-assembly of block copolymers can be precisely regulated at the nanoscale to overcome the physical limits of conventional processing techniques. This bottom-up assembly strategy is simple, easy to control, and associated with high density and high order, which is of great significance for mass transportation through membrane materials. In this review, to investigate the regulation of block copolymer self-assembly structures, we systematically explored the factors that affect the self-assembly nanostructure. After discussing the formation of nanostructures of diverse block copolymers, this review highlights block copolymer-based mass transport membranes, which play the role of “energy enhancers” in concentration cells, fuel cells, and rechargeable batteries. We firmly believe that the introduction of block copolymers can facilitate the novel energy conversion to an entirely new plateau, and the research can inform a new generation of block copolymers for more promotion and improvement in new energy applications.

## 1. Introduction

Two or more thermodynamically incompatible polymer blocks with diverse physical and chemical properties are covalently bonded together to form so-called block copolymers (BCPs) [1]. In the 1950s, the discovery of the surfactant Pluronic (PEO-b-PPO-b-PEO) [2,3,4] first attracted the attention of scientists. The mass production of BCP received a boost in 1956 due to the invention of living anionic polymerization [5]. Subsequently, other controlling polymerization techniques can also be used to synthesize BCP, such as living cationic polymerization [6], atom transfer radical polymerization (ATRP) [7,8,9,10,11], and reversible addition-fragmentation chain transfer polymerization (RAFT) [12,13]. Edwards’ self-consistent field theory (SCFT) [14] provided a premium theoretical tool for posterior simulations to explore the phase behavior of BCP. The foundations laid by these pioneers [15,16] have greatly facilitated the advancement of materials science, technology, and theory [17]. The chemical properties of various BCP blocks tend to vary with amenability to addition. To exploit the controllable properties of BCP, scientists have combined theoretical derivation with experimental practice to modify the structure of BCP for desired applications. This requires a thorough understanding of the factors that govern the nanostructure of BCP to regulate it according to the actual needs.

The BCP system can stand out and become the focus of research due to its incomparable self-assembly ability [18], and BCP can undergo accurate microphase separation in the range of 10–100 nm, which expands a new method to prepare nanodevices. The self-assembly of BCP forms rich nanostructures due to the difference in structure, block ratio, and sequences [19]. Surface structure and nanostructure are the keys to improving the properties in applications such as electronic lithography [20], organic photovoltaics [21], nanofiltration [22], and ultrafiltration [23], where electron transport or surface features dictate the performance of the devices. BCP has the potential to boost the properties of these nanodevices [24], which can result in their flourishing applications in different areas due to unique nanoscale customizable template nanostructures [25]. This review intends to highlight the effective exploitation and performance enhancement of BCP for novel energy sources instead of repeating the aforementioned applications. Our main focus will be on the construction and application of BCP-based nanostructured materials in mass transport for energy conversion. The massive exploitation and usage of nonrenewable resources, such as oil and coal, has become a burden on the environment [26]. In recent years, global warming has been responsible for frequent natural disasters [27,28], which have resulted in an irresistible surge to limit the traditional energy methods that incur huge carbon emissions and foster innovative and efficient clean energy sources. Concentration cells, fuel cells, and rechargeable batteries that can convert chemical energy into electricity without carbon emissions are new energy sources with the potential to replace traditional energy sources in the foreseeable future [29]. Using membranes for mass transportation is a key part to obtain high power efficiency from the cells of these devices related to new energy conversion processes [30], which makes the development of a high-performance battery diaphragm very important.

Because the so-called structure determines the properties, we devote a fairly lengthy section to the nanostructure of BCP before presenting their properties relevant for various practical applications. First, we briefly analyze the relevance of three vital parameters in the microphase separation of BCP. Second, various factors affect the self-assembly structure of BCP, such as the chemical structure and external conditions during the assembly process. We elaborate on the effect of various factors on the regulation of self-assembled structures, such as the chemical structure, solvent types, polymer solution concentration, nonsolvent, external field conditions, and additive aspects. Well-defined nanostructures with high order and high density can be obtained by this bottom-up self-assembly method of BCP, which can be used as nanochannel templates for mass transport. Finally, as the highlight of this paper, we investigate the application of BCP-based membranes in energy conversion, such as concentration cells, fuel cells, and rechargeable batteries. Finally, we conclude with futuristic perspectives. We hope that this review can provide a theoretical basis for how to regulate the nanostructure of polymers and offer ideas to further promote the application of block copolymers in new energy fields.

## 2. Microphase Separation of Block Copolymer

Spontaneous phase separation occurs in BCP due to repulsion between chemically and thermodynamically incompatible blocks. At the macroscopic scale, the phase separation between blocks cannot occur due to the presence of covalent bonds between the blocks of BCP [31]. Instead, in the macromolecular-length scale, phase separation can only occur at the microscopic scale, i.e., microphase separation [32]. Due to the microphase separation, there is structural arrangement of BCP at the macroscopic scale, which is known as the self-assembly phenomenon [33] and responsible for the generation of new boundaries between blocks and can result in various assembly structures.

**Figure 1:**
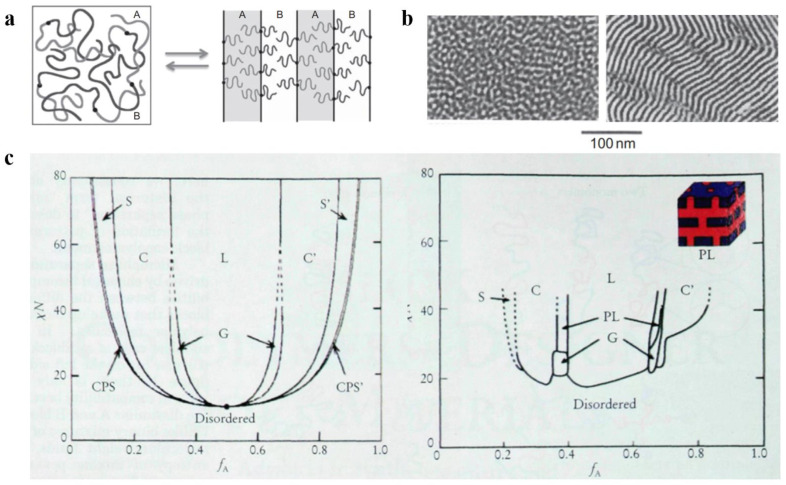
(**a**) Molecular chain distribution of AB-type diblock copolymer before and after the ODT [37]. (**b**) TEM images of PS-b-PI before and after the ODT [37]. (**c**) Theoretical phase diagrams predicted by the self-consistent field theory of the linear AB diblock copolymer and the actual phase diagram (spherical (S, S’), cylindrical (C, C’), bicontinuous gyroidal (G), and lamellar (L)) obtained from the PS-b-PI [39].

Three significant parameters are responsible for the microphase separation between the blocks: (1) the relative volume fraction *f* of each block, which is used to characterize the microscopic composition of BCP; (2) the Flory–Huggins parameter *χ*, which is used to characterize the interaction between the blocks [34,35] and is inversely proportional to the temperature change; (3) the total degree of polymerization *N* of the polymer. Among them, *χN*, which represents the phase separation strength, plays a decisive function in the separation state of BCP [36]. Increasing the temperature or decreasing *χN* gradually decreased the incompatibility between the blocks. Molecular-level mixing changes polymeric molecular chain from the stretched chain state with obvious interfaces and ordered arrangement to the disordered Gaussian chain state, as shown in Figure 1a, which results in an order-to-disorder transition (ODT) [37] of the polymer. The critical *χN* value for the occurrence of ODT is approximately 10.5. Electron microscopy can be used to clearly observe the ODT transition process of some BCPs with lower molecular weights, as shown in Figure 1b, which shows TEM images of poly(styrene)-b-poly(imide) (PS-b-PI) before and after undergoing the ODT process.

Due to the introduction of new theories such as the thermal up-and-down effect and mean-field theory, the improved self-consistent field theory (SCFT) can more accurately calculate the BCP phase behavior by scientists [38]. The theoretical phase diagram of the AB diblock copolymer [39] based on self-consistent mean-field (SCMF) theory showing the changes in phase behavior with increasing or decreasing values of *f_A_* and *χN* in a concrete manner is shown in Figure 1c (left). However, the experimental phase diagram deviates from the theoretical phase diagram because there are various practical and objective factors. Figure 1c (right) shows the experimental phase diagram of the PI-b-PS copolymer obtained by Bates et al. Both experimental and theoretical phase diagrams of PI-b-PS are qualitatively consistent with the variation occurring on the quantitative scale. First, the critical *χN* value for ODT is approximately 20, with the loss of symmetry for the phase diagram at approximately *f_A_* = 1/2. Instead of the CPS phase, a mesostable porous lamellar PL phase appears between C and L regions (calculations confirmed the mesostability of this phase later on). The difference in the PI and PS blocks from the theoretically assumed AB blocks is the main reason behind these deviations. The molecular morphology, properties, and interactions between PI and PS are responsible for the difference in experimental and theoretical values. In the experimental phase diagram, the ODT transitions can directly proceed in both the disordered D region and ordered regions that only occur at the critical point in the theoretical phase diagram, and this phenomenon can be mainly attributed to the effect of the thermal up-and-down phenomenon. With an increasing number of blocks, the phase behavior of block copolymers becomes more complicated [40,41,42].

## 3. Regulation of the Self-Assembled Structure of the Block Copolymer

### 3.1. Influence of the Molecular Structure on the Self-Assembled Structure

**Figure 2:**
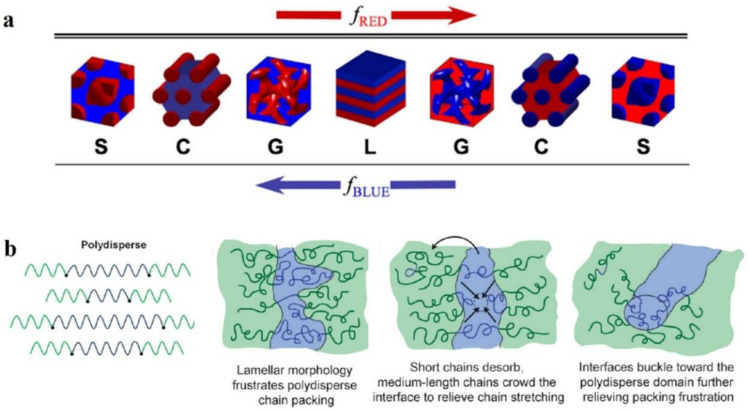
(**a**) Four equilibrium-ordered state transitions in diblock copolymers determined by relative volume fraction *f*. Spherical (S), cylindrical (C), bicontinuous gyroidal (G), and lamellar (L) [46]. (**b**) Schematic diagram of polydispersed B-blocks in ABA-type triblock copolymers leading to distortion of block microdomain interfaces [54].

The structural morphology of BCP films is our main interest. The self-assembly of BCP film is affected by many factors [43,44,45] which can be divided into molecular structure factors and assembly condition factors. The most fundamental strategy to regulate the nanoscale morphology of BCP is to adjust its molecular structure, since the BCP structure determines the self-assembly properties. Above the ODT transition curve, the thermodynamically stable nanostructures of BCP mainly consist of spherical S, cylindrical C, bicontinuous gyroidal G, and lamellar L geometries. As shown in Figure 2a, a change in volume fraction f of the AB block changes the morphology of the AB diblock copolymer [46]. Depending on whether f_A_ < 0.5 or f_B_ < 0.5, block A is dispersed within the continuous phase composed of block B or vice versa, and with increasing f_A_, the transformation of S→C→G→L occurs, while the same S’→C’→G’→L transition occurs with increasing f_B_. The size of each block in each form is determined by the molecular weight and compatibility of the blocks. With increasing molecular weight of the polymer, the blocks become segregated with increasing distance between the domains, and these factors make it challenging to undertake studies of high molecular weight BCPs [47].

The structure of BCP is also characterized by an essential parameter known as polydispersity index (PDI) [48] of molecular weight, which is calculated as PDI = M_n_/M_w_ [49]. The BCPs obtained from the polymerization of reactive anions are monodisperse [50]. However, in recent years, with the emergence of novel methods for synthesizing BCPs, most of the obtained BCPs have been polydisperse [51]. It is necessary to study the effect of broadening the molecular-weight distribution on the phase behavior of BCPs. With the concept of maintaining thermodynamic equilibrium to the maximum possible intent, Ruzette et al. [52] investigated the self-assembly behavior of triblock copolymer ABA with varying molecular-weight distributions, with narrower dispersion of block B (poly (butyl acrylate) (PBA)) and wider dispersion of block A (poly (methyl methacrylate) (PMMA)). The resulting transparent BCP membranes are homogeneous. The TEM characterization reveals the possibility of forming classical S, C, G, and L structures. However, a highly curved interface between the blocks and the PMMA block was formed mainly due to the shift of the interface to release the stretching energy of the molecular chains.

Lynd et al. [53] investigated the effect of molecular-weight distribution on the occurrence of ODT for two different BCPs prepared of polydispersed poly (ethylene-acrylamide)-b-poly (DL-propyleneglycol) (PL) and polystyrene-b-polyisoprene (SI). Ultimately, during the occurrence of ODT, increasing polydispersity in blocks with fewer (*f* < 0.5) or more (*f* > 0.5) components decrease or increase in the critical *χN* value, respectively. Widin et al. [54] reported the structural effects of a low-dispersion block polystyrene (PS) and a high-dispersion block polybutylene (PB) on the triblock copolymer SBS. As shown in Figure 2b (left), the molecular chain of highly dispersive block PB is stabilized by the low dispersive PS block in the middle position. Finally, similar experimental results to those of Ruzette were obtained, where the phase separation interface was bent toward the polydisperse segment because the polydisperse block has a smaller filling volume than the monodisperse block of the same molecular weight (the phase interface bending process is shown in Figure 2b (right)). The polydisperse B-block also reduces the critical *χN* value for ODT and increases the position of the lamellar phase window. The investigations show that by playing with the polydispersity of a particular block in BCP, the microphase separation state of BCP and its thermodynamic stability can be regulated.

### 3.2. Influence of Assembly Conditions on the Self-Assembled Structure

Normally, in addition to the interactions between polymer molecules, external film formation conditions can also affect the nanostructure. Hence, the type of solvent, polymer solution concentration, nonsolvent, additives, and external field conditions during the film formation process can affect the structure of BCP.

#### 3.2.1. Solvents to Prepare the Membranes and Self-Assembly

**Figure 3:**
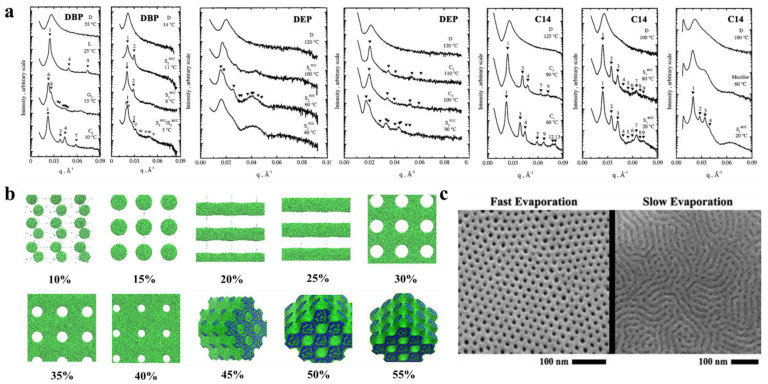
(**a**) Representative SAXS profiles as a function of temperature for DBP, DEP, and C14 solutions [55]. (**b**) Nanoscale morphology diagram of PS-b-PMMA porous membrane simulated by DPD when dissolved in tetrahydrofuran solvent at different polymer concentrations. PMMA is shown in green, and PS is shown in blue [59]. (**c**) SEM images of different morphologies formed by PS-b-PLA at varied solvent evaporation rates [62].

The selectivity of solvents toward different blocks significantly affect the self-assembled structures. Hanley et al. [55] dissolved PS-b-PI in bis(2-ethylhexyl) phthalate (DOP), which is a nonselective solvent for PS and PI, di-n-butyl phthalate (DBP), and diethyl phthalate (DEP) solvents, which are sequentially more selective for the PS block, and tetradecane (C14), which is a selective solvent for the PI blocks, to observe their phase behavior by small-angle X-ray scattering (SAXS) (Figure 3a). The PS-b-PI membrane exhibited the transformation of G→hexagonal filled C→D, which, when dissolved in different solvents, exhibited different nanostructures. After dissolution in DBP, the phase transition process of PS-b-PI was L→G’→C’→S’. The phase behavior in DEP, which is more selective for PS, like DBP, was similar to that of DBP but with higher *T_ODT_*. In contrast, in C14, for the PI selective solvent, the G’→C’→S’ ordered phases were the only occurring transitions because enhancement of segregation between microdomains by the selective solvents improve the stability of the ordered phase, which results in the appearance of more ordered phase behavior. The formation and closure of pores in BCP nanostructures can also be regulated by selective solvents [56]. The spontaneously formed poly(styrene)-b-poly (2-vinyl pyridine) (PS-b-P2VP) membrane is a dense, nonporous film. However, when dissolved in P2VP selective solvent (e.g., ethanol), the P2VP blocks dissolve to form a membrane with round or elliptical pores surrounding the core of the PS blocks. With increasing time or temperature, these pores grow into a columnar network and finally form three-dimensional interconnected pores. This pore formation process is often reversible, and immersion of the porous membrane in a PS block selective solvent (e.g., cyclohexane) closes the pores to restore the dense, nonporous membrane.

In addition, dissolution of the self-assembled structure of BCPs in mixed solvents should be explored. Yi et al. [57] investigated the nanostructure of poly(styrene)-b-poly (4-vinyl pyridine) (PS-b-P4VP) by adding it to N, N-dimethylformamide, DMF, (selective for P4VP block, and slow volatilization), tetrahydrofuran, THF, (selective for PS, faster volatilization), and their mixtures. In pure DMF solvent, a nanoscale spherical structure was obtained for PS-b-P4VP, a dense and smooth surface was obtained in pure THF, while the mixed solvent yielded a rod-like aggregated structure where the diameter of the nanospheres was similar to that observed in DMF. When polymers are dissolved in a mixture of solvents with different solubilities and volatilities, the preferential evaporation of one solvent leads to a concentration gradient within the BCP. This concentration gradient leads to phase separation of the BCP, the so-called evaporation-induced phase separation (EIPS) [58] of the solvent.

In addition, the concentration of the polymer solution is an important factor that affects the nanostructure of BCP. Jiang et al. [59] was the first to simulate the membrane formation of a double hydrophobic poly(styrene)-b-poly (methyl methacrylate) (PS-b-PMMA). The effect of the polymer concentration on the morphology of polymer molecules was also investigated in the polymer concentration range of 10–55%. Figure 3b clearly shows the difference in morphologies corresponding to different polymer concentrations. For low polymer concentrations, microphase separation just begins to occur, and the formation of a continuous phase in the system under this condition is not possible because there is not a sufficient BCP concentration, which forms a spherical structure at lower concentrations. With increasing polymer concentration, a structure can be observed because a continuous phase forms by BCP. With a further increase in concentration, porous membranes with elliptical pores start to form. When the concentration increases to approximately 30%, the elliptical pores change to circular pores. From there on, a further increase in polymer concentration only decreases the pore size within the porous membrane and membrane wall thickening. When the polymer concentration reaches approximately 45%, the polymer shows an irregular sponge-like, cross-linked structure, and finally, a continued increase in polymer concentration causes the pores to plug and results in a continuous structure of the polymer. This experiment indicates that, within a certain concentration range, the polymer concentration can be adjusted to achieve different polymer morphologies and obtain porous membranes with ideal pore size and stability.

Formation of the BCP membrane by selective solvents requires three steps: solvent uptake, swelling, and solvent evaporation drying. The effects of the first two steps on the BCP have been described in the previous section, while the evaporation rate of the solvent has been found to affect the structural orientation of BCP. It has been proven that fast solvent evaporation leads to a vertical orientation of the internal structure [60], while slower solvent evaporation rates cause parallel structural orientation or structures with mixed orientation [61]. The evaporation rate determines the propagation of phase separation from the membrane surface to the interior. Similar conclusions were obtained by Phillip et al. [62] when comparing the morphology of poly(styrene)-b-poly (D, L-lactide) (PS-b-PLA) under rapid solvent evaporation and slow evaporation (Figure 3c). Because ultrafast solvent evaporation makes steep concentration gradients rapidly propagate the vertical direction of the membrane surface, the cylindrical structure orients along the surface’s normal direction.

**Figure 4:**
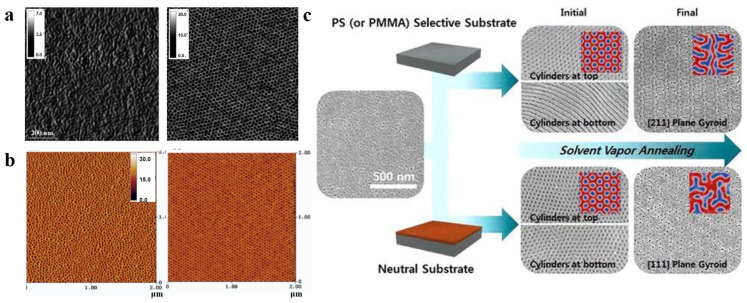
(**a**) AFM phase images (1 × 1 μm^2^) of as-cast conventional PS-b-PEO film and PS-b-PEO film annealed in PS-selective toluene vapor for 5 min [66]. (**b**) AFM phase diagrams of PS-b-PEO films obtained by spin-coating method and annealed in neutral benzene vapor for 48 h [73]. (**c**) SEM images of the (111) and (211) planes produced by solvent vapor annealing of PS-b-PMMA films in neutral substrate and selective substrate [76].

During the self-assembly process via solvent vapor annealing (SVA), the solvents are found to have a significant influence. For a given polymer system, the choice of different solvents can result in different nanostructures [63,64,65]. Peng et al. [66] investigated the effect of SVA on the morphology of poly(styrene)-b- poly (ethylene oxide) (PS-b-PEO). As shown in Figure 4a, the nanostructure of the PS-b-PEO membrane obtained by conventional casting at room temperature was a disordered and irregular worm-like morphology, while the PS-b-PEO membrane resulting from 5 min of steam annealing treatment with toluene (a selective solvent for PS) was transformed into a highly ordered arrangement of hexagonally filled perpendicular cylindrical microdomains.

However, due to preferential wetting of the surface, a thermodynamically preferred morphology should be formed with a cylindrical orientation parallel to the surface instead of perpendicular to the surface, as experimentally obtained in [67]. The formation mechanism of this morphology has not been understood, and various researchers have attempted to use computer simulations or experimental investigations to explain this phenomenon [68,69,70]. Phillip et al. [71] analyzed the evolution of the internal morphology of PS-b-PLA during SVA. Theoretical analysis and experimental verification found that the generation of such cylinders evolved from spherical intermediates nucleated at the vapor/polymer membrane interface due to the rapid evaporation of solvent, which created a concentration gradient in the BCP, and the perpendicular orientation of the intermediates with respect to the surface and epitaxial growth into cylinders. However, too-fast evaporation of the solvent does not allow sufficient time for nucleation. Lin et al. [72] used TEM analysis to demonstrate that the length of this ordered arrangement could reach the level of membrane thickness. In other words, SVA can achieve a directional ordered arrangement perpendicular to the membrane surface throughout the membrane, which is of great significance for the development of certain ion- or proton-conducting membranes or water-permeation membranes with controlled pore sizes. Kim et al. [73] demonstrated that SVA yielded a more ordered, structurally stable membrane than those obtained by conventional methods by imparting the molecular chain mobility, shielding the surface energy of the two blocks at the vapor/BCP interface and the interfacial energy between polymer and substrate, and rapidly eliminating defects in the BCP structure (Figure 4b).

When the blocks in BCP strongly interact, more variables in the SVA process will affect the structure [65,74,75]. For instance, Park et al. [76] proposed structural changes in a PS-b-PMMA membrane by choosing a selective or neutral substrate (different substrate treatments with the generated SEM images of varying morphologies are shown in Figure 4c). The membranes on neutral substrates exhibit a cylindrical structure perpendicular to the surface at the top (near the air) and bottom (near the substrate). For the SVA process on selective substrates, the top structure of the BCP membrane was unaffected, exhibiting a cylindrical structure perpendicular to the surface, but the bottom structure changed to a cylindrical structure parallel to the substrate. This result demonstrates the possibility of tailoring the pattern and structure of the BCP membrane by adjusting the membrane–substrate interaction. Cheng et al. [77] investigated various process parameters during the SVA process and ultimately demonstrated that the temperature of the substrate and vapor were also key factors in controlling the structural morphology of BCP membranes.

However, the ordered BCP membrane obtained by the selective solvent SVA method is only short-ranged. It has been shown that long-range ordering of BCP membrane in the lateral direction can be obtained via annealing with nonselective solvent (solvent with the same selectivity for each block and no specific interactions) vapors during SVA [78]. Bosworth et al. [79] obtained a long-range ordered BCP membrane by annealing poly(α-methylstyrene)-block-poly(4-hydroxystyrene) in acetone (selective solvent) vapor, which resulted in a spherical structure and tetrahydrofuran (nonselective solvent) vapor, forming a columnar phase parallel to the surface. This is due to the fact that the nonselective solvent is only uniformly distributed between the molecular chains and same selectivity for each block. It increases only their mobility without any other interactions, resulting in spontaneous formation of a cylindrical orientation parallel to the membrane plane with the lowest surface energy. SVA overcomes the shortcomings of conventional thermal annealing and enables highly ordered rearrangement of BCP in a relatively short time. By reducing the effective *T_g_*, it weakens the interactions between chains and between chains and substrates to increase the mobility of polymer chain [80].

#### 3.2.2. Nonsolvent Approaches for Constructing Nanostructure

**Figure 5:**
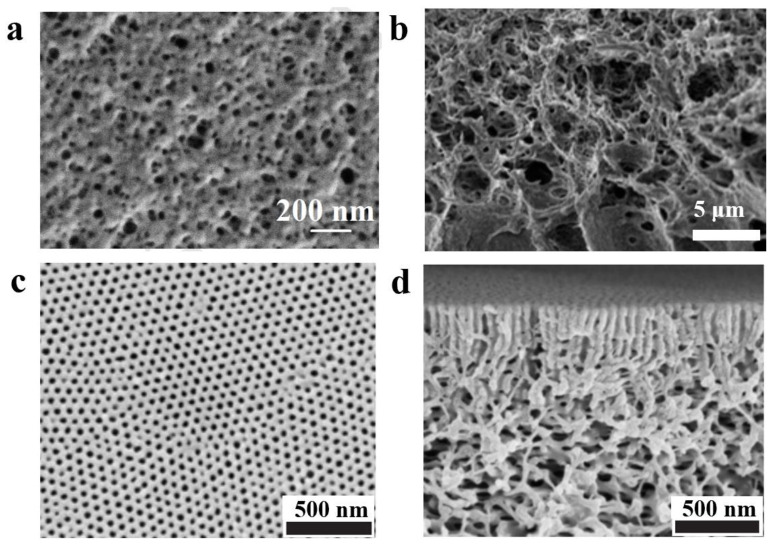
(**a**) SEM image of the surface of porous ultrafiltration membrane formed by the NIPS method for Pluronic F127 [85]. (**b**) SEM image of cross-section of multiporous sponge-like structure PI-b-PS-b-P4VP formed by NIPS method [86]. SEM images of the surface (**c**) and cross section (**d**) of PS-b-P4VP membrane obtained by SNIPS [93].

Researchers have also investigated the effect of nonsolvent-induced phase separation (NIPS) [81] of BCPs intensively [82,83,84]. The polymer is dissolved in solvent to form a homogeneous solution, followed by the slow addition of reagents which are more soluble in the solvent compared to the polymer (called extractants) to extract the solvent. The resulting two-phase structure with the polymer as continuous phase and solvent as the dispersed phase can lead to a polymer with a certain pore structure after the removal of solvent. Plisko et al. [85] modified poly (ethylene glycol)-b-poly (propylene glycol)-b-poly (ethylene glycol) (Pluronic F127) by the NIPS method employing polysulfone (PSF), and the polymer membrane obtained by the NIPS method was found to be porous in comparison to the dense polymer membrane prepared by the EIPS method (the surface structure of the porous film is shown in Figure 5a). Similarly, Gu et al. [86] fabricated porous PI-b-PS-b-P4VP membrane with sponge-like structure using the NIPS method, and Figure 5b shows the cross-sectional structure of the sponge-like porous film. NIPS is the most commonly used method for the preparation of membranes for separation [87,88], but the pore size and distribution of pores on the surface of NIPS-formed membranes are irregular and not well-controlled [89,90], and presence of some large pores can lead to material defects resulting in reduced mechanical strength of the membrane. This feature is responsible for limiting the application of BCP membranes made by the NIPS method in a great way.

In order to overcome the shortcoming mentioned above, a new NIPS strategy has emerged in the recent years: a short self-assembly is formed via short solvent evaporation after pouring the BCP into a solvent or mixed solvent, and then it was immersed in a nonsolvent for NIPS [91,92]. By this method, a monolithic, asymmetrically structured membrane, as shown in Figure 5c,d, can be formed [93]. Self-assembly results in the formation of highly ordered, dense pores in the upper thin selective layer which are uniformly perpendicular to the membrane surface [94]; pores of the lower layer which exhibit a sparse, disordered sponge-like interconnected pore channel are formed by NIPS [95]. This method of combining self-assembly (S) of BCP with nonsolvent-induced phase separation (NIPS) is named SNIPS [96,97]. As the first step of membrane formation via SNIPS is dissolution of BCP for self-assembly, different solvent parameters that affect the nanostructure (different selective solvents, mixed solvents, and solvent volatilization rate) discussed in the previous section also affect the membrane structure formed by the SNIPS method [98,99,100].

#### 3.2.3. The Influence of External Field on Self-Assembled Structure of BCP

**Figure 6:**
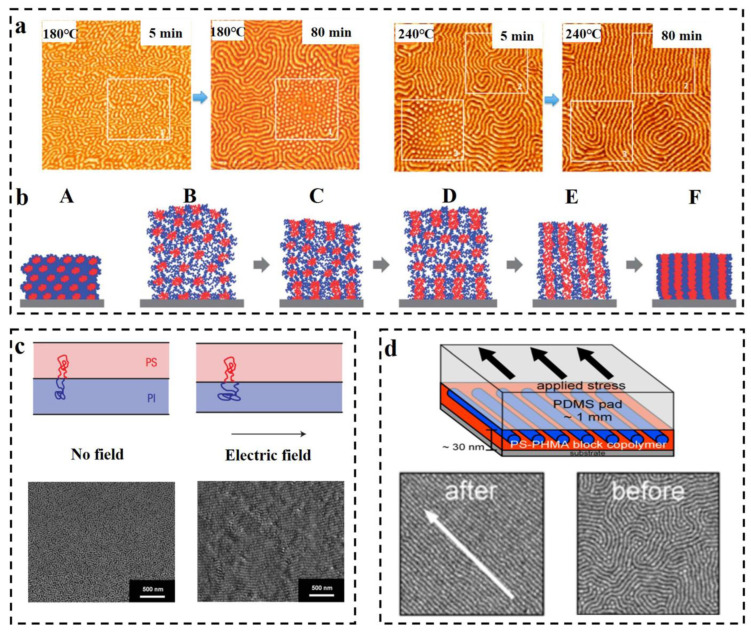
(**a**) In situ AFM images (1.25 × 1.25 μm^2^) of PS-b-PMMA obtained by thermal annealing at 180 °C and 240 °C for 5 min and 80 min, respectively [109]. (**b**) The cylindrical microdomain structure of PS-b-P4VP perpendicular to the surface is illustrated by the combination of programmed temperature rise and fall and SVA [112]. (**c**) The impact of electric field on the stretching of BCP molecular chains is illustrated and the microdomains are macroscopically oriented along the electric field direction in SEM images [116,118]. (**d**) Schematic diagram of the unidirectional shear force applied to PS-b-PHMA and TM-AFM phase images of the block nanoscale before and after shear force application. Scale bar: 500 nm [125].

Thermal annealing has proved to be one of the efficient methods for preparing long-range ordered BCP membranes [101,102,103,104], and temperature is an effective and adjustable experimental condition during the process of self-assembly. Thermal annealing refers to the heating of BCP above its glass transition temperature T_g_ [105] for allowing the molecular chains within the polymer to self-assemble to the state closest to thermodynamic equilibrium [106,107,108]. Stehlin et al. [109] used AFM to observe the morphological variation of PS-b-PMMA at 180 °C and 200 °C (well above its T_g_). Their resultant AFM images are shown in Figure 6a. It illustrates that thermal annealing results in ordered arrangement of the molecular chains, and the orderliness of PS-b-PMMA structure at 200 °C is higher than that of 180 °C for the same time, which indicates that the higher the annealing temperature, the faster the order of the arrangement. However, the BCP membrane produced by conventional thermal annealing techniques often shows structural defects, and the range of order regulation is limited, and for a thick membrane, ordering of the molecular chains over a large area cannot be obtained [110]. Moreover, for some polymers, the spacing between T_g_ and the viscous flow temperature T_f_ is small, which may not be sufficient for the rearrangement of molecular chains to take place within a short time.

In some systems, a combined method of the thermal annealing and solvent annealing methods can be used [111]. Kim et al. [112] experimentally investigated PS-b-P4VP membrane in chloroform vapor (the schematic diagram is shown in Figure 6b). Since chloroform is selective for the PS block, the PS block is pulled toward the outside of the membrane, and the P4VP block forms multilayer spherical micelles. The solvent vapor increases the mobility of the polymer molecular chains by swelling the membrane, and consequently, some of the P4VP blocks fuse. A single SVA process is insufficient to drive the fusion of P4VP spherical structures and subsequently convert them into columnar structures perpendicular to the membrane surface as a result of strong repulsion between the PS and P4VP blocks (B). During the cooling process, chloroform vapor escapes from the inside along the direction perpendicular to the membrane as the membrane shrinks, which allows the spherical structures to aggregate along the vertical direction (C). During further SVA with programmed heating, the aggregation of spherical microdomains can propagate to the inner center of the film (E), resulting in membrane expansion (D), and shrink when cooled. After several cycles of operation, vertically oriented P4VP cylindrical microdomains are obtained (F). This experiment provides an effective orientation method for the orderly arrangement, with strong interactions between the blocks or thick membrane. Furthermore, combining thermal annealing with SVA is enabled to surmount the structural uncontrollability difficulties of high molecular weight BCP, which is due to the low mobility of molecular chains caused by chain entanglement [113].

As early as 1991, Gurovich [114] made a theoretical derivation of the force and phase behavior of BCP in an electric field, but application of electric fields in the research of BCP has stopped at the theoretical level for a while since then. This delay ended when A. Bo¨ker [115] observed the morphological changes of due to the application of DC electric field on the PS-b-PI prepared via reactive anion polymerization. In the absence of an applied electric field, the PS-b-PI self-assembled as ordered lamellar structures in the control group, while under the application of an electric field, lamellar structures of the samples underwent macroscopic orientation along the electric field lines. The application of electric field achieves long-range ordered nanostructures. Schmidt [116] verified the changes in the lamellar structure of PS-b-PI with increasing strength of the electric field. The phase space parallel to the electric field direction decreases, while the phase space perpendicular to the electric field direction increases, mainly resulting from the stretching of polymer molecular chains along the electric field lines (the mechanism is shown above in the Figure 6c). Schoberth et al. [117] used quasi in situ scanning force microscopy (SFM) capable of solvent vapor treatment of BCP despite high electric fields to characterize the phase behavior. Figure 6c below shows the SFM image of the disordered Polystyrene-b-poly (2-hydroxyethyl methacrylate)-b-poly (methyl methacrylate) S_47_H_10_M_43_ oriented along the electric field direction after the application of an electric field [118]. Subsequently, it was experimentally demonstrated that application of an electric field decreases the *T_ODT_* of PS-b-PI [119].

BCP orientation can also be regulated by the application of shear fields [120]. Unidirectional shear force is applied on the BCP membrane by contacting with suitably selected moving solid surface to induce the internal structure transformation of BCP. The shear modification of polystyrene-b-polybutadiene-b-polystyrene (SBS) and the study of its rheological behavior were carried out by Morrison et al. [121]. The structure of SBS can become oriented along the direction of shear force. Angelescu et al. [122] demonstrated that the rate of shearing plays a decisive role for obtaining the molecular alignment. Low shearing rates allow the molecular chains to arrange in an ordered manner along the shear direction, while too-high shear rates may cause a disordered arrangement of the intramolecular structures. It was then shown that for similar temperature, shear orientation requires less time compared to annealing and generates structures with other defects such as disruption of grain boundaries and absence of dislocations almost in the shear range [123]. Pujari et al. [124] observed a lamellar structure perpendicular to the substrate when the self-assembly of BCP was obtained through the application of shear stress to PS-b-PMMA during annealing. Different structures of BCP such as S, C, and L structures were oriented through the application of shearing. Davis et al. [125] investigated the morphological changes of poly(styrene)-b-poly (n-hexyl methacrylate) (PS-b-PHMA) due to application of shearing and also probed the effect of shearing on different polymer composition (see Figure 6d for a schematic diagram of the apparatus for applying shear and its results). It was finally concluded that shear causes orientation along the shear direction, and that for membranes with varying PS content, the dislocation density is maximum for the membrane containing the highest amount of PS. The ordering of shear field presents a fresh approach toward nanostructural adjustment of BCP membrane, which can achieve unprecedented structural ordering at lower temperatures and under simpler conditions [126,127].

#### 3.2.4. Additives for Regulating Self-Assembly Structure

Researchers also explored various types of substances that can act as electrostatic additives [128] to regulate the nanomorphology of BCP [129], especially, to obtain some morphology in narrow interval [130]. Ionic liquid (IL) is a liquid composed of only anions and cations, with high ionic conductivity, chemical stability, and high thermal stability. Ionic liquids, when doped into BCP, can selectively complex with the charged blocks and induce lyotropic phase transitions via strong electrostatic cohesion [131]. Kim et al. [132] impregnated BCP into different ionic liquids to observe the variation in morphologies. It was demonstrated that the IL, 1-ethyl-3-methylimidazolium p-toluenesulfonate, could induce the structural transformation of the BCP from L to C morphology at a particular concentration, and different ILs result in the induction of different morphologies. Later, Bennett [133] added the IL 1-ethyl-3-methylimidazolium bis(trifluoromethanesulfonyl)imide (EMIM) to PS-b-PMMA to obtain a phase transition in the following sequence: D→L→G→C→D→S. IL-induced lyotropic phase transitions of BCP correspond to the single ion-induced phase transitions, while the role of multicharged ions on the phase transitions have rarely been reported, although it is easier to obtain strong electrostatic cohesion by multicharged ions. Polyoxometalate (POM) is a class of polymetallic oxygen cluster compounds formed by pretransition metal ions linked by oxygen, which has recently been applied for controlling the BCP nanostructures [134,135]. Zhang et al. [136] observed a change of an originally ordered columnar phase structure of the block copolymer into a disordered bicontinuous phase structure via incorporating Keggin-type POM H_4_SiW_12_O_40_ (SiW) into PS-b-P4VP, which was due to strong electrostatic cohesion between the POM and P4VP chains.

## 4. Mass Transport Applications in Energy Conversion of Block Copolymers

BCPs are one of the most important materials for the preparation of nanostructures by the bottom-up self-assembly method. Self-assembly of BCPs results in well-defined nanostructures with suitable volume fractions as well as Flory–Huggins interaction parameters. These structures can be used as nanochannels’ template for mass transport. Regulation of the functional groups and structures of the channels is possible via molecular structure designing. The channel size can be tuned between 10 to 100 nm and channel density can reach up to 10^11^ cm^−2^. High-resolution nanopatterns can be widely used in the energy field, such as sensors, memory, battery separator, etc. We concentrate our focus on mass transport applications of BCP in the energy conversion.

### 4.1. Ion Selective Membranes for Concentration Cell

Use of ion-selective membranes for converting osmotic energy into electrical energy is known as reverse electrodialysis (RED) [137,138,139,140]. Concentration cells use this energy conversion system based on salinity gradient [141,142,143]. Concentration cells are expected to solve the current energy crisis [144,145] by converting the salinity-gradient energy between seawater and river water into electricity [146]. However, the performance of conventional commercial ion-selective membranes has not been satisfactory due to their high resistance and low electrical power density. The key in developing salinity-gradient-dependent energy conversion is the preparation of high-performance ion-selective membranes [147,148]. In living organisms, ion channels are mainly composed of asymmetric membrane proteins embedded in lipid layers; these channels are capable of controlling the transport of specific ions or molecules through the membrane and also participate in various complex life activities of the human body.

Researchers have been working to imitate nature in order to find high-performance membrane materials [149]. Different shapes of nanopores comparable to lipid layers on membranes have been etched by methods known as ion-path etching [150]. Then, asymmetric nanochannels can be obtained by modifying the charge on the surface of the nanochannels [151]. Surface charge inside the inner wall of channels is the major controlling factor of ion transport [152,153]. The surface charge shows ion selectivity via adsorption of counter ions and repulsion of the co-ions [154], which can be used for salinity-gradient-driven energy conversion. Due to distinctive and controllable self-assembly properties, ion-selective membranes based on BCP have been used as separators in concentration cells [155]. Researchers carried out controlled synthesis of BCP membranes with different self-assembled structure for ion transportation. The key to improving the energy conversion efficiency of membranes mainly lies in the construction of ordered [156], high-density nanochannels having asymmetric chemical composition, pore size, and surface charges [157].

**Figure 7:**
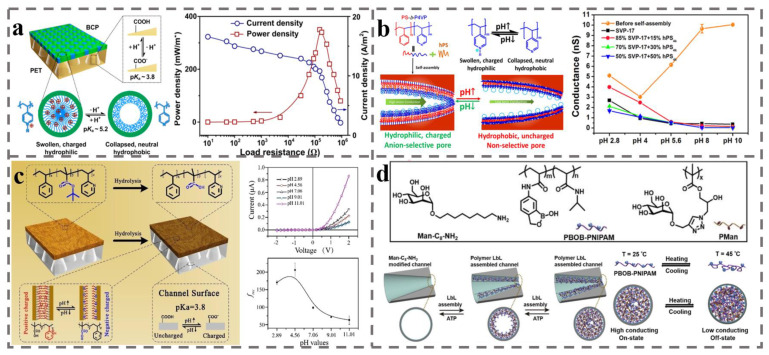
(**a**) Schematic diagram of a hybrid membrane consisting of a pH-responsive BCP membrane and a PET membrane with tapered pores obtained by ion-path etching and its permeation energy conversion efficiency [159]. (**b**) The pH-sensitive ionic conductivity of PS-b-P4VP with h-PS formed by self-assembly of molecular chain morphology with h-PS within bullet-shaped PET nanopores [160]. (**c**) The pH-responsive carboxyl groups, pyridine groups on BCP, and carboxyl groups in the PET pores enable pH-responsive, unidirectional rectification of ions in BCP-PET-laminated membrane [161]. (**d**) Molecular structures of feedstock polymers and synthetic routes to temperature-sensitive hybrid films [162].

The common approach toward construction of asymmetric nanochannels is to combine BCP with some pre-existing and well-studied nanochannels. For example, based on their excellent physical and mechanical properties and dimensional stability, polyethylene terephthalate (PET) membranes are often fabricated into nanopores of various shapes by ion-path etching [158]. Zhang et al. [159] added self-assembled positively charged PS_48400_-b-P4VP_21300_ onto the surface of negatively charged PET membrane. The structure of the hybridized membrane is shown in Figure 7a (left). PS_48400_-b-P4VP_21300_ self-assemble into highly ordered, hexagonally packed pores, and when the *pH* is below the *pKa*, the P4VP chains exhibit a swollen, positively charged, and hydrophilic state. Ion-path-etched PET membrane contains conical channels. In the hybrid membrane, nanochannels with asymmetric chemical structure, channel shape and size, and surface charge polarity were formed, and the resulting membrane showed anion selectivity and ultrahigh ion rectification (rectification ratio *f_rec_* up to 1075). The asymmetric structure of the hybrid membrane increases the energy conversion efficiency by eliminating the concentration polarization phenomenon to obtain a power density of 0.35 W m^−2^ in a 50-fold salinity gradient (Figure 7a (right)).

Wang’s group [160] introduced the strategy of layer-by-layer (LBL) self-assembly to regulate the channel size and improve the ion transport capacity of the channels. The PET membrane with bullet-shaped pores was immersed in a mixture of PS-b-P4VP and homopolymerized polystyrene (h-PS). Then, the BCP and homopolymer was bonded onto the surface of the PET membrane and the inner surface of the pores by employing the solvent annealing-induced nanowetting in the template (SAINT) method. The self-assembly of LBL on the surface of PET membrane and the inner surface of the channel improves the energy conversion efficiency by reducing the effective pore size. Furthermore, as shown in Figure 7b, pH value affected the stretching state of the P4VP chains introduced within the bullet PET channel, leading to pH-responsive behavior of the wettability, ion rectification, ionic conductivity and ion selectivity of this hybrid membrane. Previous studies have shown that the performance of the membranes in concentration cell systems is usually pH-responsive, while few systems have maintained good ion rectification even in multiple pH environments. Yang’s group [161] incorporated poly (tert-butyl methacrylate) (PtBuMA) into the PS-b-P2VP/PET hybrid nanochannels (the structure of the hybrid membrane is shown in Figure 7c (left)), which hydrolyzes in alkaline solution to produce negative charges, while the pyridine group in the P2VP block provide positive charges in acidic conditions. The membrane maintained a high *f_rec_* (up to 200) with changing pH values (its rectification properties are shown in Figure 7c (right)). Similarly, Wu’s group [162] employed the LBL method to graft thermally responsive PBOB-b-PNIPAM (with negative charge) into PET conical pores (schematic diagram of the synthesis process is shown in Figure 7d). The *f_rec_* increased from 1.64 to 10.66 by introduction of the polymer into the PET hole, while increasing the number of layers resulted in decreasing conductivity and *f_rec_*. As wettability and molecular conformation of PBOB-b-PNIPAM in channels depend on temperature, selective change of ion rectification and conductivity of the channels can be obtained by changing the temperature.

In addition, one-dimensional (1D) and two-dimensional (2D) hybrid membranes can also be obtained when BCP is composited with 2D layered nanochannels. 2D nanomaterials have attracted the interest of researchers due to their simplicity of preparation, ease of controlling their structure and components, and possibility to extend in a large scale [163]. The unique layered stacking structure of 2D nanomaterials possesses large specific surface area and can be easily modified with functional groups. For example, MXene, with intrinsic functional groups between its layers, has received wide interest in the application of concentration cell [164]. Lin et al. [165], in an innovative method, hybridized negatively charged MXene membrane with positively charged PS-b-P2VP membrane into inhomogeneous membrane containing asymmetric structure, components, and charges. The ion selectivity experiment indicated dominant anionic selectivity for the BCP membrane with respect to MXene. This hybridization resulted in a power density of 6.74 W m^−2^ at pH = 11.

**Figure 8:**
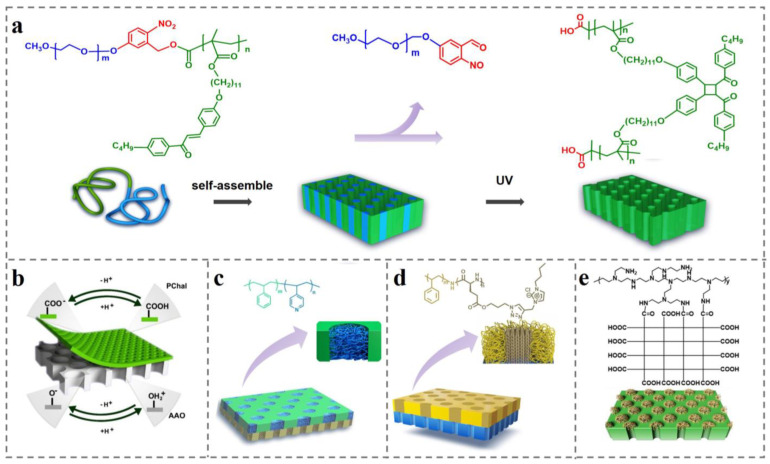
(**a**) Structural formula of PEO-hv-PChal molecule which can be degraded and cross-linked simultaneously under UV light and its self-assembly process [166]. (**b**) Schematic diagram of BCP-AAO asymmetric nanochannel membrane structure [166]. (**c**) Schematic diagram of PS-b-P4VP nanopore channel and schematic diagram of asymmetrical ultrathin Janus film hybridized with PEO-b-PChal porous membrane [168]. (**d**) Schematic diagram of the nanopore channels of PS-b-PPLG and its hybridization with PEO-b-PChal porous membrane to form a membrane with asymmetric nanopore channels [169]. (**e**) Schematic diagram of the molecular chain structure of PEO-b-PChal and polyethyleneimine and their hybridization to form mushroom-like asymmetric nanopore channels [170].

Beside these commercial BCPs, a newly designed functional BCP has also been developed. Our group [166] synthesized a UV-sensitive PEO-hv-PChal by ATRP. Introduction of o-nitrobenzyl ester as a degradable group between the two blocks resulted in breakage of this group, leading to the in situ formation of carboxylic acid groups under ultraviolet light. Self-supporting ordered nanochannels can be prepared by one-step degradation and cross-linking under the same UV light by employing PEO with good solubility as the degradation removal phase and the liquid crystal molecule containing chalone group as the continuous phase (Figure 8a). The size, density, and degree of order of the channels can be controlled by molecular weight and dispersion of the BCP. Then, we constructed asymmetric nanochannels by compounding the ordered nanochannels with AAO membrane. AAO is used by researchers in various fields of research because of its precise, stable, and un-deformed honeycomb structure with uniform pore size distribution and adjustable aperture. This asymmetric organic–inorganic hybrid membrane owned different channels and groups. Solution pH controls the ionization states of both the carboxyl group in the PChal and the hydroxyl group in the AAO pore (the pH-influenced groups within the hybridized pore channel are shown in Figure 8b). High energy conversion efficiency can be achieved over a wide pH range due to the synergistic effect of the hybrid nanochannels [167].

Although the above hybridization methods of combining BCP with other organic or inorganic membranes are able to achieve high rectification ratios or high power densities, further improvement of their performance is limited by the high internal resistance of the membrane (membrane thicknesses are often only in the micron range) and weak adhesion between the heterogeneous membranes. This motivated researchers to search for a strategy that can achieve high conversion efficiency with low internal resistance of the membrane. Zhang et al. [168] achieved innovative hybridization of two BCP membranes composed of different structural units (PEO-b-PChal and PS-b-P4VP) into an ultrathin ion-selective Janus membrane having only 500 nm thickness (the structure is shown in Figure 8c). This Janus membrane achieved excellent ion rectification due to asymmetric chemical structure, geometric aperture structure, and opposite surface charges. The results show that the P4VP chain in the channel of PS-b-P4VP membrane plays a dominant role in ion selectivity, making the hybrid membrane anion-selective. However, the operating conditions of the Janus membrane depend on the state of P4VP chains, as the performance of Janus membrane is mainly affected by the P4VP chains. Finally, this hybrid membrane was able to achieve a power density of 2.04 W m^−2^ in a seawater–river water environment at pH = 4.3.

However, the ion transport of BCP-based nanochannels is hindered due the resulting disorder and embedding of functional group distribution originating from disorder due to chaotic nature of the random coil conformation of P4VP flexible chain in the range of 1–10 nm, which makes them easy to unwind. This is a limiting factor for the further improvement of BCP-based nanochannels’ performance. Our group [169] further proposed hybridization of rigid rod-like-structured PS-b-PPLG onto the prepared PEO-b-PChal porous membrane. Poly (γ-benzyl-L-glutamate) (PBLG) is a poly(polypeptide) with stable α-helical rigid structure, and its branched chain makes it easier to modify with ionic liquids as functional groups. After the introduction of ionic liquid imidazole bromide on the α-helical rigid-structured PBLG to provide positive charges, this hybrid membrane possesses nanochannels with asymmetric surface charge and nanostructure (the structure of the asymmetric nanochannel membrane is shown in Figure 8d). The rigid PPLG segments exist in a straight chain conformation in the channel, and the arrangement is ordered in the size range of 1–10 nm, forming hierarchical ordered structures with different sizes. This method provides a strategy to improve the orderliness of the channel and reduce the internal resistance of nanochannels.

To further improve the power density of energy conversion, Li et al. [170] demonstrated a robust mushroom-shaped (with stem and cap) nanochannel array membrane with an ultrathin selective layer and ultrahigh pore density. The stem parts, negative-charged 1D channels, are prepared from the previous PEO-b-PChal self-assembly with a density of ~10^11^ cm^−2^, while the cap parts, positive-charged 3D channels, are formed by chemically grafted hyperbranched polyethyleneimine. As shown in Figure 8e, the hyperbranched polyethyleneimine cap parts as the selective layer are equivalent to tens of 1D nanochannels per stem. These robust mushroom-shaped nanochannels achieve *f_rec_* of 17.3 and power density of 15.4 W m^−2^ at a KCl solution with 500-fold salinity gradient.

The SNIPS method also gives a new perspective for the preparation of monolayer asymmetric membrane. Koo et al. [171] combined SNIPS with a thermodynamic method for controlling the growth process of nanochannels in the membrane to achieve an asymmetric porous PS-b-P2VP membrane. The porous BCP membrane formed by the SNIPS method can spontaneously form asymmetric nanopore channels with dense nanopores on the surface and spongy and looser micropores inside. Parameters such as membrane thickness can also be controlled by thermodynamic methods. In addition, the P2VP channels can be made positively charged by quaternization, which imparts anion selectivity into the membrane and reduces the pore dimensions. Connecting a large number of salinity-gradient cells with PS-P2VP membranes can be used for the operation of small electronic devices.

Xie et al. [172] exploited a new method for the utilization of BCP in the construction of nanochannels: the nanochannels can be regulated by utilizing and modifying the different chemical properties of each block of BCP as a template. Different hydrophilic properties of PEO and PPO in triblock copolymer Pluronic F127 were used as soft templates to synthesize 1D anisotropic carbon-ordered mesoporous nanowires (CMWs). Then, dense CMWs membranes (with negative charge) were synthesized on porous AAO membranes (with positive charge) by the vacuum filtration method. In the CMWs membrane, ions can pass through the 3D interconnected channels formed by the gaps of these CMWs. Therefore, the prepared asymmetric hybrid membranes possess asymmetric chemical components, nanostructures, and surface charges, and the membrane exhibited excellent cation selectivity.

### 4.2. Ion Exchange Membranes for Fuel Cell

A fuel cell is a device capable of converting chemical energy from fuels (such as methanol, ethanol, pure hydrogen, natural gas, and gasoline) and directly oxidizing it into electrical energy through an electrochemical reaction [173,174]. It has the advantages of high efficiency, no toxic gas emission, and no pollution, and is regarded as the most promising way for generation of electricity [175,176]. For example, in hydrogen fuel cell, the basic principle is the reverse reaction of water electrolysis, where hydrogen and oxygen are supplied to the anode and cathode, respectively. After outward diffusion of hydrogen through the anode, it reacts with the electrolyte to release electrons which then reach the cathode through external load. Electrolyte diaphragm is an important part of fuel cells [177]. Its main function is to conduct ions while keeping the oxidizer and reducing agent separate [178]. The ion transport occurs through the groups present in the membrane-forming material, through the combination and separation of ions, and via forming a strip of ion channels [179]. For example, proton-exchange membranes usually have some strong electrolyte groups such as sulfonic acid radical that can easily bond with the protons [180]. Protons can easily bond with the groups and release, allowing the protons to pass through the membrane to form a current without direct contact between the positive and negative oxidizers and the fuel. Similarly, the function of cation-exchange membrane [181] and anion-exchange membrane is to allow the cations or anions to pass through, forming a current, while blocking positive and negative oxidizer and fuel. The principle is based on the selective permeability of ion-exchange membranes [182].

A proton-exchange membrane fuel cell is a new type of fuel cell with high efficiency and low pollution [183,184]. Proton-exchange membrane (PEM) [185], also known as polymer electrolyte membrane, is a very thin, rigid, plastic-like polymer material with proton conductivity that can be used to replace conventional liquid electrolytes [186,187]. The biggest challenge of PEM is to improve ionic conductivity and thermal stability without compromising the mechanical strength [188,189,190]. Inspired by the common BCP structures having rigid blocks as the skeletal support and flexible blocks as ion conductors, BCPs are usually used as precursors for nanostructured polymer materials [191].

**Figure 9:**
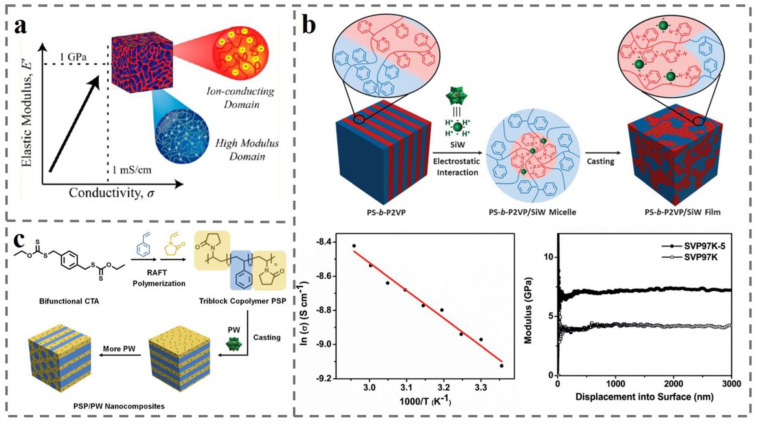
(**a**) BMITFSI induces PS-b-PEO to form a bicontinuous phase microdomain structure and enhances the elastic modulus *E*’ and conductivity *σ* of PS-b-PEO [194]. (**b**) The electrostatic interaction between SiW and PS-b-P2VP induces the formation of a bicontinuous phase favorable to proton conduction, leading to an increase in proton conductivity and Young’s modulus [195]. (**c**) PW induces PVP-b-PS-b-PVP by electrostatic interaction to form a proton-conducting continuous phase with cylindrical, mechanically enhanced phase with great proton-conduction advantage [196].

The bicontinuous phase of self-assembled BCP has attracted much attention. There are two separate and mixed structures, the domain is throughout the material structure, it has a very high interfacial area and connectivity, and it is advantageous to the ion transport in the membrane. However, bicontinuous phase in the phase diagram has a relatively narrow interval, and it is difficult to achieve by controlling the molecular weight and proportion. Doping of other molecules to regulate the interaction forces is a good strategy to regulate the self-assembled structure of BCP. Electrostatic interaction between IL [192,193] and BCP is a promising pathway to induce BCP to form a bicontinuous phase. Morgan et al. [194] used 1-butyl-3-methylimidazolium bis (trifluoromethylsulfonyl) imide (BMITFSI) to induce the self-assembly of AB-type diblock copolymer PS-b-PEO. BMITFSI is immiscible with PS, and it enters the PEO block to induce the separation of PS-b-PEO assembly into a bicontinuous phase (the bicontinuous phase structure is shown in Figure 9a). Additionally, as shown in Figure 9a, IL also reduces the intermolecular forces between the polymer chains to enhance the fluidity of polymer chain, improves the ionic transport property of the membrane, and affects the free volume and flexibility of the membrane, improving the conductivity and electrochemical stability of the membrane.

It has also been found that in spite of IL, POM is also used to induce BCP to establish a bicontinuous phase structure. Zhang et al. [195] induced the transition of an AB-type diblock copolymer PS-b-P2VP from layered structure to bicontinuous phase structure using H_4_SiW_12_O_40_ (SiW) (the structural transformation process is shown in the top diagram of Figure 9b). POM in BCP plays the role of electrostatic morphology control system, improves the plasma conductivity, and also plays the role of nanointensifier for improving the modulus. Ultimately, as shown in the lower graph of Figure 9b, POM increased the proton conductivity *σ* and Young’s modulus of the bicontinuous structured material at room temperature by 0.1 mS cm^−1^ and by 7.4 G Pa, respectively. Zhai et al. [196] introduced H_3_PW_12_O_40_ (PW) into the synthesized ABA-type triblock copolymer PVP-b-PS-b-PVP. As shown in Figure 9c, weak electrostatic interactions between PW and PVP units induced the BCP to form a three-dimensional continuous-charged domain (for ionic conduction) and an inverse cylindrical phase structure with embedded neutral domains (for mechanical support). The nanocomposite corresponding to this configuration was shown to be a novel PEM with proton conductivity *σ* of 1.32 mS cm^−1^.

The alkaline fuel cell composed of anion exchange membrane (AEM) is similar to the PEM fuel cell in terms of mechanism and complete reaction equilibrium [197], but the electrode reaction is different from that of the PEM fuel cell, as the reaction takes place under alkaline conditions. Alkaline fuel cells have distinct advantages: the cost of fuel cell production can be reduced by some inexpensive catalysts such as iron and nickel [198,199]; liquid fuels such as methanol and ethanol that can be stored and transported easily are used [200]; and the corrosion of metal catalysts is less than that of acidic environment, prolonging the fuel cell life [201]. AEMs are the critical components of alkaline fuel cells. Their ionic conductivity and stability under alkaline environments are critical to the performance of alkaline fuel cells [202,203]. Microstructure of the polymer determines the material performance, and the design of a customized AEM with a rational structure is essential for the production of high-performance alkaline fuel cells [204,205].

**Figure 10:**
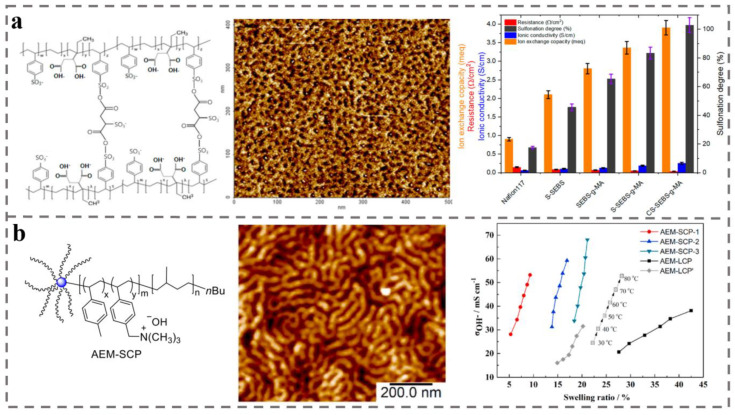
(**a**) Molecular structure formula of S-SEBS-g-MA AEM and its AFM phase image. Compared with AEM formed by grafting different molecular chains and Nafion 117, S-SEBS-g-MA AEM has the superior ionic conductivity and *IEC* performance [209]. (**b**) Molecular structure and AFM image of AEM-SCP membrane and its anion conductivity versus swelling ratio [212].

Sulfonated poly (styrene-ethylene-butylene-styrene) copolymer (SEBS) is a promising diaphragm material, having high thermal and chemical stability, adjustable mechanical properties, and good proton conductivity and cost-effectiveness [206,207,208]. S-SEBS-g-MA AEM was prepared by grafting sulfonic acid and maleic anhydride to form ion channel for improvement of ion conductivity [209] (Figure 10a). From electrochemical analysis, as shown in Figure 10a (right), the modified membranes showed increased ionic conductivity, ionic exchange capacity (*IEC*), and water absorption, all of which were higher than those of the conventional commercial Nafion 117 membrane. These results suggest that modified AEMs based on BCPs such as SEBS can have comparable potential as AEMs with respect to the commercial Nafion 117.

Polyolefin-based AEMs exhibit good alkali resistance and have great potential for mass production due to their easy processability and low cost [210,211]. In the anionic polymerization, the chemical composition, molecular weight, and conformational distribution of the products can be controlled during the process of polymer synthesis. Polyisoprene-b-poly(4-methylstyrene) (SCP) AEMs with a star topology and improved properties were prepared (see Figure 10b left diagram for SCP-AEM structural formula) through anionic polymerization by Pan et al [212]. This star-shaped structure is a head-to-head cross-linked structure. The conductivity and low water absorption and alkaline stability of AEM can be significantly improved through functionalization such as bromination and quaternary ammonium near the “star-shaped”, nucleus resulting in the construction of continuous ion transport channels (AFM phase diagram is shown in Figure 10b (middle)). The *IEC* and hydroxide conductivity of the functionalized AEM can reach 2.15 mmol g^−1^ and 68.1 mS cm^−1^, respectively. With increasing current density, maximum power density of the fuel cell reaches 120.2 mW cm^−2^. This method provides a new approach to enhance the performance of AEM by taking advantage of the customizable structural properties of BCP. The conductivity and basic stability of AEM can be improved by placing the functional groups that can build continuous ion channels and the chain segments that are easily attacked by hydroxide in the inside structure of BCP by structural design.

### 4.3. Battery Separator for Rechargeable Batteries (e.g., Lithium-Ion Batteries)

Among commercially implemented rechargeable batteries, high specific energy and stable cycling performance have resulted in the broad applications of lithium-ion batteries (LIBs) [213,214] in cell phones, notebook computers, electric vehicles, and grid energy storage, with prospects for further development. However, the performance and safety of lithium-ion batteries still need improvement [215,216]. As a key component, lithium ions transfer between the positive and negative electrodes during the charging and discharging process and are controlled by a battery separator, which in turn controls the efficiency of the lithium-ion battery [217,218]. An important parameter characterizing the migration of lithium ions is *t+*, which is defined as the ratio of Li^+^ migration rate to that of the migration of all ions in the electrolyte for a lithium-ion battery [219,220]. In liquid electrolytes, *t+* is normally below 1, with the general value being between 0.3 and 0.4 [221]. Traditional LIBs commonly used organic solvent-resistant, high-strength porous polyolefin membrane as a battery separator, which has good chemical stability and mechanical stability, but the thermal stability is poor. During heat build-up and rise of temperature inside the battery, the diaphragm can melt, which results in a short-circuit between the positive and negative terminals, causing an accident [222]. In recent years, there have been frequent safety accidents concerning LIBs, which seriously threaten the safety of human life and limit the application prospects of LIBs. Researchers are committed to finding ways to improve the safety of LIBs [223,224], especially by enhancing the thermal stability of the battery diaphragm to prevent accidents.

**Figure 11:**
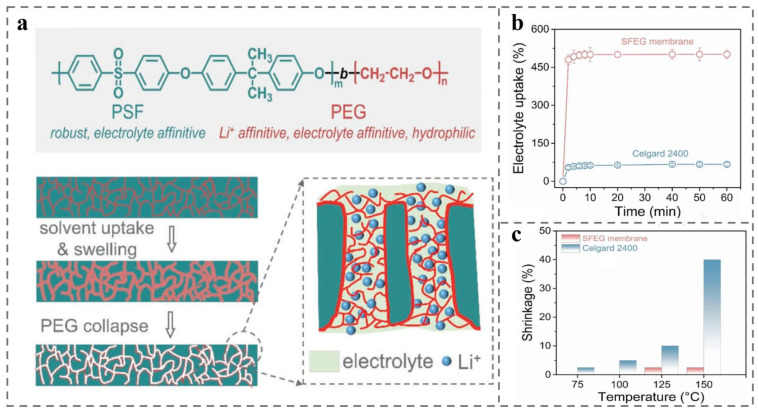
(**a**) Molecular structure of PSF-b-PEG membrane and its selective swelling application in lithium-ion transport. Compared to the Celgard 2400 diaphragm, PSF-b-PEG membrane exhibits exceptional electrolyte uptake performance (**b**) and thermal stability (**c**) [225].

Researchers have employed polymer membranes with higher melting points and good thermal stability, such as polyimide, cellulose, and PSF, to replace traditional polyolefin materials, which has shown primary results regarding enhancement of thermal stability of LIBs. Yang et al. [225] studied PSF-b-PEG, a BCP composed of PSF and hydrophilic polyethylene glycol PEG, as a diaphragm for LIBs (diaphragm structure and lithium-ion transport process are shown in Figure 11a). This BCP has the following advantages: high thermal stability of PSF, strong affinity for the liquid organic electrolytes, and good complexation ability of PEG toward lithium salts, allowing for transport of lithium ions, which greatly improves the lithium-ion conductivity. In addition, presence of benzene ring and ether bond on the PSF chain of PSF-b-PEG provides rigidity and flexibility, performing good mechanical strength. A strong interaction force between the two blocks results in good mechanical stability for PSF-b-PEG during the formation of membrane and channels, resulting in no ion channel blockage. As is shown at Figure 11b,c, PSF-b-PEG has been tested to be comparable to commercial polypropylene Celgard 2400 membranes, both with respect to thermal stability and porosity at 380 °C, but the PSF-b-PEG membranes have additional advantages of much higher wettability, mechanical strength, and electrolyte absorption than Celgard 2400. During the tests in the temperature range of 75 °C to 150 °C, PSF-b-PEG membranes consistently maintained relatively low thermal shrinkage, ensuring the safety of LIBs. Additionally, at temperatures higher than 125 °C, the channels of PSF-b-PEG membrane will automatically close due to thermal annealing, switching “off” the working state of the battery diaphragm, and during the process of channels closure, there will not be any change of membrane size, ensuring the safety of LIBs.

Even after using thermally stable diaphragms, safety accidents cannot be eliminated at the root, and liquid organic electrolytes still have safety hazards [226]. Solid polymer lithium-ion batteries, which use solid polymer electrolytes (SPE) instead of traditional liquid electrolytes [227], are safer than traditional liquid LIBs and will gradually become the mainstream of LIBs by replacing the traditional liquid LIBs [228,229]. The ion channels of SPE should be perpendicular to the electrode surface to facilitate ion transport [230]. BCP electrolytes (BCPEs) are one of the most attractive alternatives to traditional liquid electrolytes in LIBs, as they can improve thermal and mechanical stability while maintaining ionic conductivity.

**Figure 12:**
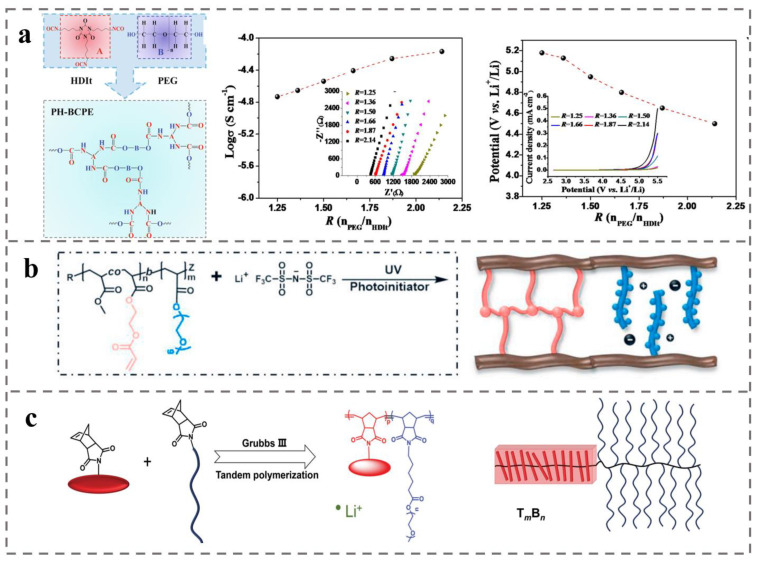
(**a**) PEG and HDIt molecules were copolymerized to obtain PH-BCPE with 3D network structure. *R* has a decisive effect on the ionic conductivity and electrochemical window of PH-BCPE [231]. (**b**) Synthesis of P(DBEA-co-MA)-b-PEG with 3D cross-linked network structure [232]. (**c**) TB-BCPEs were synthesized by tandem polymerization and have a lamellar structure favorable for ion transport [233].

In general, rigid blocks are used as the skeleton support to provide mechanical strength, whereas flexible blocks are used to enhance ionic conductivity. PEG has been widely used in LIBs but has associated problems such as low ionic conductivity, poor mechanical strength, and narrow electrochemical window, which can be improved by reintroducing rigid blocks. Lin et al. [231] utilized cross-linking copolymerization reactions of flexible PEG blocks and rigid hexamethylene di-isocyanate trimer (HDIt) blocks in different ratios to synthesize a series of new BCPEs, named PH-BCPE, with 3D networks (see Figure 12a (left)). It has been experimentally demonstrated that the ratio of two blocks *R* (*n_PEG_*/*n_HDIt_*) can be a controlling factor for the performance of PH-BCPE. By increasing the proportion of flexible PEG blocks (elevation of *R* value), the ionic conductivity of PH-BCPE can be increased (as shown in Figure 12a (middle)) and the interfacial resistance with the electrode can be decreased, whereas increasing the proportion of rigid HDIt blocks (decrease in *R* value) can improve the electrochemical window (as shown in Figure 12a (right)) and mechanical strength of PH-BCPE. Finally, the ionic conductivity of obtained PH-BCPE resulted in an ionic conductivity up to 5.7·10^−4^ S cm^−1^, *t+* value of 0.49, and wide electrochemical window up to 4.65 V (vs. Li^+^/Li).

To achieve a BCPE with high mechanical stability and ionic conductivity, He et al. [232] employed RAFT polymerization to synthesize a difunctional P(DBEA-co-MA)-b-PEG which consists of the UV-cross-linkable block P(DBEA-co-MA) and suspended PEG chains (the network structure is shown in Figure 12b). The mechanical strength can be improved due to the presence of tethered double bonds in P(DBEA-co-MA) which can form a cross-linked network, while the suspended PEG molecular chains with low-crystallinity can increase molecular mobility and reduce chain entanglement to improve ionic conductivity. The interfacial resistance between the electrode and P(DBEA-co-MA)-b-PEG-SPE can be reduced from 5500 Ω cm^2^ to 100 Ω cm^2^ compared to the conventional PEO-SPE. The cycle stability of the battery is doubled, and the battery can be cycled for more than 700 h at 22 °C, showing significant improvement of durability. Using bis (trifluoromethane) sulfonimide lithium salt (LITFSI) as the ion carrier, the final value of *t+* was calculated to be 0.35.

Among the various forms of BCP, cylindrical (C) pore channels have been applied, though the lamellar (L) structures possess higher *f* values and theoretically higher ionic conductivity than C, but L has been explored very little. Liu et al. [233] employed an intermediate layer of azobenzene to synthesize a series of tablet-b-bottlebrush (TB) BCP electrolytes (Figure 12c). The liquid crystal polymer segments can form nanopores in a directional and rapid manner after solvent thermal annealing and the presence of polyethylene oxide (PEO) side chains leads to increasing electrical conductivity. The performance of electrolyte membrane with a thickness of 200 mm or more prepared by this method is superior to other spin-coated nanoscale membrane. The final synthesized SPE also possesses an ionic conductivity of 2.19 mS cm^−1^ at 200 °C, along with superior thermal stability.

## 5. Conclusions

The self-assembly behaviors of BCP can be regulated by mastering the molecular structure, solvent type, concentration of polymer solution, nonsolvent, external environmental conditions, and additives. Additionally, the self-assembly behaviors of BCP can precisely control the structure of BCP at the nanoscale. Moreover, due to their unparalleled surface-controllable and nanostructure-controllable properties, BCP can be introduced into a broad spectrum of applications. This review covers advancements in block copolymers for the optimization of energy conversion in novel batteries. The integration of BCP into new energy sources such as concentration cells, fuel cells, and lithium-ion batteries can improve energy conversion efficiency while sustaining high performance, which is unachievable with classical materials.

In spite of the attractive options provided by BCP in enhancing energy conversion efficiency, for new energy devices, there is still considerable work to be done to make them competitive with existing technologies in terms of performance and cost. The first challenge is to utilize novel materials for the synthesis of block copolymers with new and superior structures and properties. The materials currently used to synthesize block copolymers are conventional polymeric materials, and it is critical to explore new materials as well as new structures for BCPs. This becomes potentially changing as more applications are discovered. The second challenge is how to precisely tune the interface during self-assembly to tailor BCPs with absolutely precise nanostructures on demand. In many cases, different disciplines are not well-integrated, and perhaps the collision of different sciences will bring chances for precise tuning of BCPs. Finally, due to the constraints of the established technology platform and cost issues, the research on BCP-based composites is still at the primary stage of laboratory research rather than large-scale commercial application. This requires not only a change in technology, but also a long process of financial investment and training of personnel. Certainly, all of these bottlenecks are common barriers to the implementation of new technologies, and the future is still bright with the endless possibilities of BCP. We are convinced that block copolymers can enable a new era of energy conversion; the question is not if, but when.

## Data Availability

Not applicable.

## References

[1] Schacher F.H., Rupar P.A., Manners I. (2012). Functional Block Copolymers: Nanostructured Materials with Emerging Applications. Angew. Chem. Int. Ed..

[2] Bates C.M., Bates F.S. (2017). 50th Anniversary Perspective: Block Polymers-Pure Potential. Macromolecules.

[3] Mankowich A.M. (1954). Micellar Molecular Weights of Selected Surface Active Agents. J. Phys. Chem..

[4] Vaughn T.H., Suter H.R., Lundsted L.G., Kramer M.G. (1951). Properties of some newly developed nonionic detergents. J. Am. Oil. Chem. Soc..

[5] Szwarc M., Levy M., Milkovich R. (1956). Polymerization initiated by electron transfer to monomer. A new method of formation of block polymers. J. Am. Chem. Soc..

[6] Lanson D., Ariura F., Schappacher M., Borsali R., Deffieux A. (2007). Application of living ionic polymerizations to the design of AB-type comb-like copolymers of various topologies and organizations. Macromol. Res..

[7] Charleux B., Delaittre G., Rieger J., D’Agosto F. (2012). Polymerization-Induced Self-Assembly: From Soluble Macromolecules to Block Copolymer Nano-Objects in One Step. Macromolecules.

[8] Nasrullah M.J., Vora A., Webster D.C. (2011). Block Copolymer Synthesis via a Combination of ATRP and RAFT Using Click Chemistry. Macromol. Chem. Phys..

[9] Siegwart D.J., Oh J.K., Matyjaszewski K. (2012). ATRP in the design of functional materials for biomedical applications. Prog. Polym. Sci..

[10] Matyjaszewski K. (2018). Advanced Materials by Atom Transfer Radical Polymerization. Adv. Mater..

[11] Keddie D.J. (2014). A guide to the synthesis of block copolymers using reversible-addition fragmentation chain transfer (RAFT) polymerization. Chem. Soc. Rev..

[12] Eggers S., Eckert T., Abetz V. (2018). Double thermoresponsive block-random copolymers with adjustable phase transition temperatures: From block-like to gradient-like behavior. J. Polym. Sci. Pol. Chem..

[13] Gyorgy C., Hunter S.J., Girou C., Derry M.J., Armes S.P. (2020). Synthesis of poly(stearyl methacrylate)-poly(2-hydroxypropyl methacrylate) diblock copolymer nanoparticles via RAFT dispersion polymerization of 2-hydroxypropyl methacrylate in mineral oil. Polym. Chem..

[14] Edwards S.F. (1965). The statistical mechanics of polymers with excluded volume. Proc. Phys. Soc..

[15] Matsen M.W., Schick M. (1994). Stable and unstable phases of a diblock copolymer melt. Phys. Rev. Lett..

[16] Matsen M.W., Bates F.S. (1996). Unifying weak- and strong-segregation block copolymer theories. Macromolecules.

[17] Spencer R.K.W., Matsen M.W. (2018). Fluctuation effects in blends of A plus B homopolymers with AB diblock copolymer. J. Chem. Phys..

[18] Kang S., Kim G.-H., Park S.-J. (2022). Conjugated Block Copolymers for Functional Nanostructures. Acc. Chem. Res..

[19] Rosler A., Vandermeulen G.W.M., Klok H.A. (2012). Advanced drug delivery devices via self-assembly of amphiphilic block copolymers. Adv. Drug Deliv. Rev..

[20] Cummins C., Lundy R., Walsh J.J., Ponsinet V., Fleury G., Morris M.A. (2020). Enabling future nanomanufacturing through block copolymer self-assembly: A review. Nano Today.

[21] Meng L., Fan H., Lane J.M.D., Qin Y. (2020). Bottom-Up Approaches for Precisely Nanostructuring Hybrid Organic/Inorganic Multi-Component Composites for Organic Photovoltaics. MRS Adv..

[22] Yu H.Z., Qiu X.Y., Moreno N., Ma Z.W., Calo V.M., Nunes S.P., Peinemann K.V. (2015). Self-Assembled Asymmetric Block Copolymer Membranes: Bridging the Gap from Ultra- to Nanofiltration. Angew. Chem. Int. Ed..

[23] Radjabian M., Abetz V. (2020). Advanced porous polymer membranes from self-assembling block copolymers. Prog. Polym. Sci..

[24] Cheng X.Q., Wang Z.X., Jiang X., Li T.X., Lau C.H., Guo Z.H., Ma J., Shao L. (2018). Towards sustainable ultrafast molecular-separation membranes: From conventional polymers to emerging materials. Prog. Mater. Sci..

[25] Park H.B., Kamcev J., Robeson L.M., Elimelech M., Freeman B.D. (2017). Maximizing the right stuff: The trade-off between membrane permeability and selectivity. Science.

[26] Khan I., Hou F.J., Le H.P. (2021). The impact of natural resources, energy consumption, and population growth on environmental quality: Fresh evidence from the United States of America. Sci. Total Environ..

[27] Schiermeier Q. (2011). Increased flood risk linked to global warming. Nature.

[28] Zandalinas S.I., Fritschi F.B., Mittler R. (2021). Global Warming, Climate Change, and Environmental Pollution: Recipe for a Multifactorial Stress Combination Disaster. Trends Plant Sci..

[29] Fan E.S., Li L., Wang Z.P., Lin J., Huang Y.X., Yao Y., Chen R.J., Wu F. (2020). Sustainable Recycling Technology for Li-Ion Batteries and Beyond: Challenges and Future Prospects. Chem. Rev..

[30] Orilall M.C., Wiesner U. (2011). Block copolymer based composition and morphology control in nanostructured hybrid materials for energy conversion and storage: Solar cells, batteries, and fuel cells. Chem. Soc. Rev..

[31] Bates F.S. (1991). Polymer-polymer phase behavior. Sci.-New York.

[32] Wong C.K., Qiang X.L., Muller A.H.E., Groschel A.H. (2020). Self-Assembly of block copolymers into internally ordered microparticles. Prog. Polym. Sci..

[33] Feng H.B., Lu X.Y., Wang W.Y., Kang N.G., Mays J.W. (2017). Block Copolymers: Synthesis, Self-Assembly, and Applications. Polymers.

[34] Farrell R.A., Fitzgerald T.G., Borah D., Holmes J.D., Morris M.A. (2009). Chemical Interactions and Their Role in the Microphase Separation of Block Copolymer Thin Films. Int. J. Mol. Sci..

[35] Bates C.M., Seshimo T., Maher M.J., Durand W.J., Cushen J.D., Dean L.M., Blachut G., Ellison C.J., Willson C.G. (2012). Polarity-Switching Top Coats Enable Orientation of Sub-10-nm Block Copolymer Domains. Science.

[36] Hagita K., Aoyagi T., Abe Y., Genda S., Honda T. (2021). Deep learning-based estimation of Flory–Huggins parameter of A–B block copolymers from cross-sectional images of phase-separated structures. Sci. Rep..

[37] Yoshida H., Takenaka M., Gronheid R., Nealey P. (2015). 1-Physics of block copolymers from bulk to thin films. Directed Self-Assembly of Block Co-Polymers for Nano-Manufacturing.

[38] Liu M.J., Qiang Y.C., Li W.H., Qiu F., Shi A.C. (2016). Stabilizing the Frank-Kasper Phases via Binary Blends of AB Diblock Copolymers. Acs Macro Lett..

[39] Bates F.S., Fredrickson G.H. (1999). Block copolymers-Designer soft materials. Phys. Today.

[40] Groschel A.H., Muller A.H.E. (2015). Self-assembly concepts for multicompartment nanostructures. Nanoscale.

[41] Wanka G., Hoffmann H., Ulbricht W. (1994). Phase-Diagrams and aggregation behavior of poly(oxyethylene)-poly(oxypropylene)-poly(oxyethylene) triblock copolymers in aqueous-solutions. Macromolecules.

[42] Bates F.S., Hillmyer M.A., Lodge T.P., Bates C.M., Delaney K.T., Fredrickson G.H. (2012). Multiblock Polymers: Panacea or Pandora’s Box?. Science.

[43] Majewski P.W., Yager K.G. (2016). Rapid ordering of block copolymer thin films. J. Phys.-Condes. Matter.

[44] Abetz V., Kremer K., Muller M., Reiter G. (2019). Functional Macromolecular Systems: Kinetic Pathways to Obtain Tailored Structures. Macromol. Chem. Phys..

[45] Cui H.G., Chen Z.Y., Zhong S., Wooley K.L., Pochan D.J. (2007). Block copolymer assembly via kinetic control. Science.

[46] Lynd N.A., Meuler A.J., Hillmyer M.A. (2008). Polydispersity and block copolymer self-assembly. Prog. Polym. Sci..

[47] Hadziioannou G., Skoulios A. (1982). Molecular weight dependence of lamellar structure in styrene isoprene two- and three-block copolymers. Macromolecules.

[48] Carrot C., Guillet J. (1997). From dynamic moduli to molecular weight distribution: A study of various polydisperse linear polymers. J. Rheol..

[49] Llorens J., Rude E., Marcos R.M. (2003). Polydispersity index from linear viscoelastic data: Unimodal and bimodal linear polymer melts. Polymer.

[50] Levy M. (2007). The impact of the concept of “Living Polymers” on material science. Polym. Adv. Technol..

[51] Nguyen H.T., Tran T.T., Nguyen-Thai N.U. (2018). Preparation of polydisperse polystyrene-block-poly(4-vinyl pyridine) synthesized by TEMPO-mediated radical polymerization and the facile nanostructure formation by self-assembly. J. Nanostruct. Chem..

[52] Ruzette A.V., Tence-Girault S., Leibler L., Chauvin F., Bertin D., Guerret O., Gerard P. (2006). Molecular disorder and mesoscopic order in polydisperse acrylic block copolymers prepared by controlled radical polymerization. Macromolecules.

[53] Lynd N.A., Hillmyer M.A. (2007). Effects of polydispersity on the order-disorder transition in block copolymer melts. Macromolecules.

[54] Widin J.M., Schmitt A.K., Schmitt A.L., Im K., Mahanthappa M.K. (2012). Unexpected Consequences of Block Polydispersity on the Self-Assembly of ABA Triblock Copolymers. J. Am. Chem. Soc..

[55] Hanley K.J., Lodge T.P., Huang C.-I. (2000). Phase Behavior of a Block Copolymer in Solvents of Varying Selectivity. Macromolecules.

[56] Wang Y. (2016). Nondestructive Creation of Ordered Nanopores by Selective Swelling of Block Copolymers: Toward Homoporous Membranes. Acc. Chem. Res..

[57] Yi Z., Zhang P.-B., Liu C.-J., Zhu L.-P. (2016). Symmetrical Permeable Membranes Consisting of Overlapped Block Copolymer Cylindrical Micelles for Nanoparticle Size Fractionation. Macromolecules.

[58] Gohil J.M., Choudhury R.R., Thomas S., Pasquini D., Leu S.-Y., Gopakumar D.A. (2019). Chapter 2-Introduction to Nanostructured and Nano-enhanced Polymeric Membranes: Preparation, Function, and Application for Water Purification. Nanoscale Materials in Water Purification.

[59] Jiang H., Chen T., Chen Z., Huo J., Zhang L., Zhou J. (2020). Computer simulations on double hydrophobic PS-b-PMMA porous membrane by non-solvent induced phase separation. Fluid Phase Equilib..

[60] Paradiso S.P., Delaney K.T., Garcia-Cervera C.J., Ceniceros H.D., Fredrickson G.H. (2014). Block Copolymer Self Assembly during Rapid Solvent Evaporation: Insights into Cylinder Growth and Stability. Acs Macro Lett..

[61] Russell T.P., Coulon G., Deline V.R., Miller D.C. (1989). Characteristics of the surface-induced orientation for symmetric diblock PS/PMMA copolymers. Macromolecules.

[62] Phillip W.A., O’Neill B., Rodwogin M., Hillmyer M.A., Cussler E.L. (2010). Self-Assembled Block Copolymer Thin Films as Water Filtration Membranes. ACS Appl. Mater. Interfaces.

[63] Tseng Y.H., Lin Y.L., Ho J.H., Chang C.T., Fan Y.C., Shen M.H., Chen J.T. (2021). Reversible and tunable morphologies of amphiphilic block copolymer nanorods confined in nanopores: Roles of annealing solvents. Polymer.

[64] Lee S., Cheng L.-C., Gadelrab K.R., Ntetsikas K., Moschovas D., Yager K.G., Avgeropoulos A., Alexander-Katz A., Ross C.A. (2018). Double-Layer Morphologies from a Silicon-Containing ABA Triblock Copolymer. ACS Nano.

[65] Chavis M.A., Smilgies D.M., Wiesner U.B., Ober C.K. (2015). Widely Tunable Morphologies in Block Copolymer Thin Films Through Solvent Vapor Annealing Using Mixtures of Selective Solvents. Adv. Funct. Mater..

[66] Peng J., Han Y.C., Knoll W., Kim D.H. (2007). Development of nanodomain and fractal morphologies in solvent annealed block copolymer thin films. Macromol. Rapid Commun..

[67] Stenbock-Fermor A., Rudov A.A., Gumerov R.A., Tsarkova L.A., Boker A., Moller M., Potemkin I.I. (2014). Morphology-Controlled Kinetics of Solvent Uptake by Block Copolymer Films in Nonselective Solvent Vapors. Acs Macro Lett..

[68] Tsarkova L. (2012). Distortion of a Unit Cell versus Phase Transition to Nonbulk Morphology in Frustrated Films of Cylinder-Forming Polystyrene-b-polybutadiene Diblock Copolymers. Macromolecules.

[69] Cavicchi K.A., Berthiaume K.J., Russell T.P. (2005). Solvent annealing thin films of poly(isoprene-b-lactide). Polymer.

[70] Xuan Y., Peng J., Cui L., Wang H., Li B., Han Y. (2004). Morphology Development of Ultrathin Symmetric Diblock Copolymer Film via Solvent Vapor Treatment. Macromolecules.

[71] Phillip W.A., Hillmyer M.A., Cussler E.L. (2010). Cylinder Orientation Mechanism in Block Copolymer Thin Films Upon Solvent Evaporation. Macromolecules.

[72] Lin Z.Q., Kim D.H., Wu X.D., Boosahda L., Stone D., LaRose L., Russell T.P. (2002). A rapid route to arrays of nanostructures in thin films. Adv. Mater..

[73] Kim S.H., Misner M.J., Xu T., Kimura M., Russell T.P. (2004). Highly oriented and ordered arrays from block copolymers via solvent evaporation. Adv. Mater..

[74] Kim K., Park S., Kim Y., Bang J., Park C., Ryu D.Y. (2016). Optimized Solvent Vapor Annealing for Long-Range Perpendicular Lamellae in PS-b-PMMA Films. Macromolecules.

[75] Li X., Peng J., Wen Y., Kim D.H., Knoll W. (2007). Morphology change of asymmetric diblock copolymer micellar films during solvent annealing. Polymer.

[76] Park S., Kim Y., Lee W., Hur S.-M., Ryu D.Y. (2017). Gyroid Structures in Solvent Annealed PS-b-PMMA Films: Controlled Orientation by Substrate Interactions. Macromolecules.

[77] Cheng X., Boker A., Tsarkova L. (2019). Temperature-Controlled Solvent Vapor Annealing of Thin Block Copolymer Films. Polymers.

[78] Gowd E.B., Koga T., Endoh M.K., Kumar K., Stamm M. (2014). Pathways of cylindrical orientations in PS-b-P4VP diblock copolymer thin films upon solvent vapor annealing. Soft Matter.

[79] Bosworth J.K., Paik M.Y., Ruiz R., Schwartz E.L., Huang J.Q., Ko A.W., Smilgies D.M., Black C.T., Ober C.K. (2008). Control of self-assembly of lithographically patternable block copolymer films. Acs Nano.

[80] Arias-Zapata J., Bohme S., Garnier J., Girardot C., Legrain A., Zelsmann M. (2016). Ultrafast Assembly of PS-PDMS Block Copolymers on 300 mm Wafers by Blending with Plasticizers. Adv. Funct. Mater..

[81] Guillen G.R., Ramon G.Z., Kavehpour H.P., Kaner R.B., Hoek E.M.V. (2013). Direct microscopic observation of membrane formation by nonsolvent induced phase separation. J. Membr. Sci..

[82] Wang D.-M., Lai J.-Y. (2013). Recent advances in preparation and morphology control of polymeric membranes formed by nonsolvent induced phase separation. Curr. Opin. Chem. Eng..

[83] Guillen G.R., Pan Y., Li M., Hoek E.M.V. (2011). Preparation and Characterization of Membranes Formed by Nonsolvent Induced Phase Separation: A Review. Ind. Eng. Chem. Res..

[84] Tan X., Li K. (2011). Inorganic hollow fibre membranes in catalytic processing. Curr. Opin. Chem. Eng..

[85] Plisko T.V., Penkova A.V., Burts K.S., Bildyukevich A.V., Dmitrenko M.E., Melnikova G.B., Atta R.R., Mazur A.S., Zolotarev A.A., Missyul A.B. (2019). Effect of Pluronic F127 on porous and dense membrane structure formation via non-solvent induced and evaporation induced phase separation. J. Membr. Sci..

[86] Gu Y., Wiesner U. (2015). Tailoring Pore Size of Graded Mesoporous Block Copolymer Membranes: Moving from Ultrafiltration toward Nanofiltration. Macromolecules.

[87] Abed M.R.M., Kumbharkar S.C., Groth A.M., Li K. (2012). Ultrafiltration PVDF hollow fibre membranes with interconnected bicontinuous structures produced via a single-step phase inversion technique. J. Membr. Sci..

[88] Gin D.L., Noble R.D. (2011). Designing the Next Generation of Chemical Separation Membranes. Science.

[89] Li D.F., Chung T.S., Ren J.Z., Wang R. (2004). Thickness dependence of macrovoid evolution in wet phase-inversion asymmetric membranes. Ind. Eng. Chem. Res..

[90] Widjojo N., Chung T.S. (2006). Thickness and air gap dependence of macrovoid evolution in phase-inversion asymmetric hollow fiber membranes. Ind. Eng. Chem. Res..

[91] Abetz V. (2015). Isoporous Block Copolymer Membranes. Macromol. Rapid Commun..

[92] Foroutani K., Ghasemi S.M., Pourabbas B. (2021). Molecular tailoring of polystyrene-block-poly (acrylic acid) block copolymer toward additive-free asymmetric isoporous membranes via SNIPS. J. Membr. Sci..

[93] Peinemann K.V., Abetz V., Simon P.F.W. (2007). Asymmetric superstructure formed in a block copolymer via phase separation. Nat. Mater..

[94] Karunakaran M., Nunes S.P., Qiu X.Y., Yu H.Z., Peinemann K.V. (2014). Isoporous PS-b-PEO ultrafiltration membranes via self-assembly and water-induced phase separation. J. Membr. Sci..

[95] Stegelmeier C., Filiz V., Abetz V., Perlich J., Fery A., Ruckdeschel P., Rosenfeldt S., Forster S. (2014). Topological Paths and Transient Morphologies during Formation of Mesoporous Block Copolymer Membranes. Macromolecules.

[96] Jung A., Rangou S., Abetz C., Filiz V., Abetz V. (2012). Structure Formation of Integral Asymmetric Composite Membranes of Polystyrene-block-Poly(2-vinylpyridine) on a Nonwoven. Macromol. Mater. Eng..

[97] Rahman M.M. (2021). Selective Swelling and Functionalization of Integral Asymmetric Isoporous Block Copolymer Membranes. Macromol. Rapid Commun..

[98] Dorin R.M., Phillip W.A., Sai H., Werner J., Elimelech M., Wiesner U. (2014). Designing block copolymer architectures for targeted membrane performance. Polymer.

[99] Rangou S., Buhr K., Filiz V., Clodt J.I., Lademann B., Hahn J., Jung A., Abetz V. (2014). Self-organized isoporous membranes with tailored pore sizes. J. Membr. Sci..

[100] Radjabian M., Abetz C., Fischer B., Meyer A., Abetz V. (2017). Influence of Solvent on the Structure of an Amphiphilic Block Copolymer in Solution and in Formation of an Integral Asymmetric Membrane. Acs Appl. Mater. Interfaces.

[101] Tan K.W., Jung B., Werner J.G., Rhoades E.R., Thompson M.O., Wiesner U. (2015). Transient laser heating induced hierarchical porous structures from block copolymer-directed self-assembly. Science.

[102] Seshimo T., Maeda R., Odashima R., Takenaka Y., Kawana D., Ohmori K., Hayakawa T. (2016). Perpendicularly oriented sub-10-nm block copolymer lamellae by atmospheric thermal annealing for one minute. Sci. Rep..

[103] Albalak R.J., Thomas E.L., Capel M.S. (1997). Thermal annealing of roll-cast triblock copolymer films. Polymer.

[104] Tong Q.Q., Zheng Q., Sibener S.J. (2014). Alignment and Structural Evolution of Cylinder-Forming Diblock Copolymer Thin Films in Patterned Tapered-Width Nanochannels. Macromolecules.

[105] Sepe A., Hoppe E.T., Jaksch S., Magerl D., Zhong Q., Perlich J., Posselt D., Smilgies D.M., Papadakis C.M. (2011). The effect of heat treatment on the internal structure of nanostructured block copolymer films. J. Phys.-Condes. Matter.

[106] Shi L.-Y., Yin C., Zhou B., Xia W., Weng L., Ross C.A. (2021). Annealing Process Dependence of the Self-Assembly of Rod–Coil Block Copolymer Thin Films. Macromolecules.

[107] Majewski P.W., Yager K.G. (2015). Latent Alignment in Pathway-Dependent Ordering of Block Copolymer Thin Films. Nano Lett..

[108] Wang H.S., Kim K.H., Bang J. (2019). Thermal Approaches to Perpendicular Block Copolymer Microdomains in Thin Films: A Review and Appraisal. Macromol. Rapid Commun..

[109] Stehlin F., Diot F., Gwiazda A., Dirani A., Salaun M., Zelsmann M., Soppera O. (2013). Local Reorganization of Diblock Copolymer Domains in Directed Self-Assembly Monitored by in Situ High-Temperature AFM. Langmuir.

[110] Majewski P.W., Yager K.G. (2016). Reordering transitions during annealing of block copolymer cylinder phases. Soft Matter.

[111] Zhou Z.P., Liu G.L. (2017). Controlling the Pore Size of Mesoporous Carbon Thin Films through Thermal and Solvent Annealing. Small.

[112] Kim S., Jeon G., Heo S.W., Kim H.J., Kim S.B., Chang T., Kim J.K. (2013). High aspect ratio cylindrical microdomains oriented vertically on the substrate using block copolymer micelles and temperature-programmed solvent vapor annealing. Soft Matter.

[113] Kim E., Ahn H., Park S., Lee H., Lee M., Lee S., Kim T., Kwak E.-A., Lee J.H., Lei X. (2013). Directed Assembly of High Molecular Weight Block Copolymers: Highly Ordered Line Patterns of Perpendicularly Oriented Lamellae with Large Periods. ACS Nano.

[114] Gurovich E. (1994). On Microphase Separation of Block Copolymers In an Electric Field: Four Universal Classes. Macromolecules.

[115] Boker A., Elbs H., Hansel H., Knoll A., Ludwigs S., Zettl H., Urban V., Abetz V., Muller A.H.E., Krausch G. (2002). Microscopic mechanisms of electric-field-induced alignment of block copolymer microdomains. Phys. Rev. Lett..

[116] Schmidt K., Schoberth H.G., Ruppel M., Zettl H., Hansel H., Weiss T.M., Urban V., Krausch G., Boker A. (2008). Reversible tuning of a block-copolymer nanostructure via electric fields. Nat. Mater..

[117] Schoberth H.G., Olszowka V., Schmidt K., Boker A., Muller A.H.E., Schmidt H.W. (2010). Effects of Electric Fields on Block Copolymer Nanostructures. Complex Macromolecular Systems I.

[118] Olszowka V., Hund M., Kuntermann V., Scherdel S., Tsarkova L., Boker A. (2009). Electric Field Alignment of a Block Copolymer Nanopattern: Direct Observation of the Microscopic Mechanism. Acs Nano.

[119] Schoberth H.G., Pester C.W., Ruppe M., Urban V.S., Boker A. (2013). Orientation-Dependent Order-Disorder Transition of Block Copolymer Lamellae in Electric Fields. Acs Macro Lett..

[120] Marencic A.P., Chaikin P.M., Register R.A. (2012). Orientational order in cylinder-forming block copolymer thin films. Phys. Rev. E.

[121] Morrison F., Le Bourvellec G., Winter H.H. (1987). Flow-induced structure and rheology of a triblock copolymer. J. Appl. Polym. Sci..

[122] Angelescu D.E., Waller J.H., Adamson D.H., Deshpande P., Chou S.Y., Register R.A., Chaikin P.M. (2004). Macroscopic orientation of block copolymer cylinders in single-layer films by shearing. Adv. Mater..

[123] Angelescu D.E., Waller J.H., Register R.A., Chaikin P.M. (2005). Shear-induced alignment in thin films of spherical nanodomains. Adv. Mater..

[124] Pujari S., Keaton M.A., Chaikin P.M., Register R.A. (2012). Alignment of perpendicular lamellae in block copolymer thin films by shearing. Soft Matter.

[125] Davis R.L., Chaikin P.M., Register R.A. (2014). Cylinder Orientation and Shear Alignment in Thin Films of Polystyrene-Poly(n-hexyl methacrylate) Diblock Copolymers. Macromolecules.

[126] Marencic A.P., Adamson D.H., Chaikin P.M., Register R.A. (2010). Shear alignment and realignment of sphere-forming and cylinder-forming block-copolymer thin films. Phys. Rev. E.

[127] Kim S.Y., Nunns A., Gwyther J., Davis R.L., Manners I., Chaikin P.M., Register R.A. (2014). Large-Area Nanosquare Arrays from Shear-Aligned Block Copolymer Thin Films. Nano Lett..

[128] Wang X.J., Goswami M., Kumar R., Sumpter B.G., Mays J. (2012). Morphologies of block copolymers composed of charged and neutral blocks. Soft Matter.

[129] Sing C.E., Zwanikken J.W., de la Cruz M.O. (2014). Electrostatic control of block copolymer morphology. Nat. Mater..

[130] Goswami M., Sumpter B.G., Mays J. (2010). Controllable stacked disk morphologies of charged diblock copolymers. Chem. Phys. Lett..

[131] Zhou H., Liu C.G., Gao C.Q., Qu Y.Q., Shi K.Y., Zhang W.Q. (2016). Polymerization-Induced Self-Assembly of Block Copolymer Through Dispersion RAFT Polymerization in Ionic Liquid. J. Polym. Sci. Pol. Chem..

[132] Kim S.Y., Yoon E., Joo T., Park M.J. (2011). Morphology and Conductivity in Ionic Liquid Incorporated Sulfonated Block Copolymers. Macromolecules.

[133] Bennett T.M., Jack K.S., Thurecht K.J., Blakey I. (2016). Perturbation of the Experimental Phase Diagram of a Diblock Copolymer by Blending with an Ionic Liquid. Macromolecules.

[134] Cui T., Li X., Dong B., Li X., Guo M., Wu L., Li B., Li H. (2019). Janus onions of block copolymers via confined self-assembly. Polymer.

[135] He Q.B., Zhang Y.J., Li H.L., Chen Q. (2020). Rheological Properties of ABA-Type Copolymers Physically End-Cross-Linked by Polyoxometalate. Macromolecules.

[136] Zhang L.Y., Liu C., Shang H.Y., Cao X., Chai S.C., Chen Q., Wu L.X., Li H.L. (2016). Electrostatic tuning of block copolymer morphologies by inorganic macroions. Polymer.

[137] Guo W., Cao L.X., Xia J.C., Nie F.Q., Ma W., Xue J.M., Song Y.L., Zhu D.B., Wang Y.G., Jiang L. (2010). Energy Harvesting with Single-Ion-Selective Nanopores: A Concentration-Gradient-Driven Nanofluidic Power Source. Adv. Funct. Mater..

[138] Logan B.E., Elimelech M. (2012). Membrane-based processes for sustainable power generation using water. Nature.

[139] Wang W., Hao J., Sun Q., Zhao M., Liu H., Li C., Sui X. (2022). Carbon nanofibers membrane bridged with graphene nanosheet and hyperbranched polymer for high-performance osmotic energy harvesting. Nano Res..

[140] Siria A., Bocquet M.L., Bocquet L. (2017). New avenues for the large-scale harvesting of blue energy. Nat. Rev. Chem..

[141] Mei Y., Tang C.Y.Y. (2018). Recent developments and future perspectives of reverse electrodialysis technology: A review. Desalination.

[142] Kingsbury R.S., Chu K., Coronell O. (2015). Energy storage by reversible electrodialysis: The concentration battery. J. Membr. Sci..

[143] van Egmond W.J., Saakes M., Porada S., Meuwissen T., Buisman C.J.N., Hamelers H.V.M. (2016). The concentration gradient flow battery as electricity storage system: Technology potential and energy dissipation. J. Power Sources.

[144] Zaffora A., Culcasi A., Gurreri L., Cosenza A., Tamburini A., Santamaria M., Micale G. (2020). Energy Harvesting by Waste Acid/Base Neutralization via Bipolar Membrane Reverse Electrodialysis. Energies.

[145] Tedesco M., Cipollina A., Tamburini A., Micale G. (2017). Towards 1 kW power production in a reverse electrodialysis pilot plant with saline waters and concentrated brines. J. Membr. Sci..

[146] Siria A., Poncharal P., Biance A.L., Fulcrand R., Blase X., Purcell S.T., Bocquet L. (2013). Giant osmotic energy conversion measured in a single transmembrane boron nitride nanotube. Nature.

[147] Ma H., Wang S., Yu B., Sui X., Shen Y., Cong H. (2021). Bioinspired nanochannels based on polymeric membranes. Sci. China Mater..

[148] Li C., Jiang H., Liu P., Zhai Y., Yang X., Gao L., Jiang L. (2022). One Porphyrin Per Chain Self-Assembled Helical Ion-Exchange Channels for Ultrahigh Osmotic Energy Conversion. J. Am. Chem. Soc..

[149] Xiao K., Wen L.P., Jiang L. (2016). Biomimetic Solid-State Nanochannels: From Fundamental Research to Practical Applications. Small.

[150] Laucirica G., Albesa A.G., Toimil-Molares M.E., Trautmann C., Marmisolle W.A., Azzaroni O. (2020). Shape matters: Enhanced osmotic energy harvesting in bullet-shaped nanochannels. Nano Energy.

[151] Chen W., Xiang Y., Kong X.-Y., Wen L. (2022). Polymer-based membranes for promoting osmotic energy conversion. Giant.

[152] Emmerich T., Vasu K.S., Nigues A., Keerthi A., Radha B., Siria A., Bocquet L. (2022). Enhanced nanofluidic transport in activated carbon nanoconduits. Nat. Mater..

[153] Lin C.Y., Combs C., Su Y.S., Yeh L.H., Siwy Z.S. (2019). Rectification of Concentration Polarization in Mesopores Leads To High Conductance Ionic Diodes and High Performance Osmotic Power. J. Am. Chem. Soc..

[154] Wu Y.D., Qian Y.C., Niu B., Chen J.J., He X.F., Yang L.S., Kong X.Y., Zhao Y.F., Lin X.B., Zhou T. (2021). Surface Charge Regulated Asymmetric Ion Transport in Nanoconfined Space. Small.

[155] Sharon D., Bennington P., Dolejsi M., Webb M.A., Dong B.X., de Pablo J.J., Nealey P.F., Patel S.N. (2020). Intrinsic Ion Transport Properties of Block Copolymer Electrolytes. Acs Nano.

[156] DuChanois R.M., Porter C.J., Violet C., Verduzco R., Elimelech M. (2021). Membrane Materials for Selective Ion Separations at the Water-Energy Nexus. Adv. Mater..

[157] Wang J., Hou J., Zhang H.C., Tian Y., Jiang L. (2018). Single Nanochannel-Aptamer-Based Biosensor for Ultrasensitive and Selective Cocaine Detection. Acs Appl. Mater. Interfaces.

[158] Apel P.Y. (2019). Fabrication of functional micro- and nanoporous materials from polymers modified by swift heavy ions. Radiat. Phys. Chem..

[159] Zhang Z., Kong X.Y., Xiao K., Liu Q., Xie G.H., Li P., Ma J., Tian Y., Wen L.P., Jiang L. (2015). Engineered Asymmetric Heterogeneous Membrane: A Concentration-Gradient-Driven Energy Harvesting Device. J. Am. Chem. Soc..

[160] Wang J., Liu L., Yan G.L., Li Y.C., Gao Y., Tian Y., Jiang L. (2021). Ionic Transport and Robust Switching Properties of the Confined Self-Assembled Block Copolymer/Homopolymer in Asymmetric Nanochannels. ACS Appl. Mater. Interfaces.

[161] Yang L.S., Liu P., Zhu C.C., Zhao Y.Y., Yuan M.M., Kong X.Y., Wen L.P., Jiang L. (2021). Ion transport regulation through triblock copolymer/PET asymmetric nanochannel membrane: Model system establishment and rectification mapping. Chin. Chem. Lett..

[162] Wu Y.F., Yang G., Lin M.C., Kong X.Y., Mi L., Liu S.Q., Chen G.S., Tian Y., Jiang L. (2019). Continuously Tunable Ion Rectification and Conductance in Submicrochannels Stemming from Thermoresponsive Polymer Self-Assembly. Angew. Chem. Int. Ed..

[163] Macha M., Marion S., Nandigana V.V.R., Radenovic A. (2019). 2D materials as an emerging platform for nanopore-based power generation. Nat. Rev. Mater..

[164] Hao J., Wang W., Zhao J., Che H., Chen L., Sui X. (2022). Construction and application of bioinspired nanochannels based on two-dimensional materials. Chin. Chem. Lett..

[165] Lin X.B., Liu P., Xin W.W., Teng Y.F., Chen J.J., Wu Y.D., Zhao Y.F., Kong X.Y., Jiang L., Wen L.P. (2021). Heterogeneous MXene/PS-b-P2VP Nanofluidic Membranes with Controllable Ion Transport for Osmotic Energy Conversion. Adv. Funct. Mater..

[166] Sui X., Zhang Z., Zhang Z.Y., Wang Z.W., Li C., Yuan H., Gao L.C., Wen L.P., Fan X., Yang L.J. (2016). Biomimetic Nanofluidic Diode Composed of Dual Amphoteric Channels Maintains Rectification Direction over a Wide pH Range. Angew. Chem. Int. Ed..

[167] Sui X., Zhang Z., Li C., Gao L.C., Zhao Y., Yang L.J., Wen L.P., Jiang L. (2019). Engineered Nanochannel Membranes with Diode-like Behavior for Energy Conversion over a Wide pH Range. Acs Appl. Mater. Interfaces.

[168] Zhang Z., Sui X., Li P., Xie G.H., Kong X.Y., Xiao K., Gao L.C., Wen L.P., Jiang L. (2017). Ultrathin and Ion-Selective Janus Membranes for High-Performance Osmotic Energy Conversion. J. Am. Chem. Soc..

[169] Hao J.L., Yang T., He X.L., Tang H.Y., Sui X. (2021). Hierarchical nanochannels based on rod-coil block copolymer for ion transport and energy conversion. Giant.

[170] Li C., Wen L.P., Sui X., Cheng Y.R., Gao L.C., Jiang L. (2021). Large-scale, robust mushroom-shaped nanochannel array membrane for ultrahigh osmotic energy conversion. Sci. Adv..

[171] Koo J.M., Park C.H., Yoo S., Lee G.W., Yang S.Y., Kim J.H., Yoo S.I. (2021). Selective ion transport through three-dimensionally interconnected nanopores of quaternized block copolymer membranes for energy harvesting application. Soft Matter.

[172] Xie L., Zhou S., Liu J.R., Qiu B.L., Liu T.Y., Liang Q.R., Zheng X.Z., Li B., Zeng J., Yan M. (2021). Sequential Superassembly of Nanofiber Arrays to Carbonaceous Ordered Mesoporous Nanowires and Their Heterostructure Membranes for Osmotic Energy Conversion. J. Am. Chem. Soc..

[173] Sazali N., Salleh W.N.W., Jamaludin A.S., Razali M.N.M. (2020). New Perspectives on Fuel Cell Technology: A Brief Review. Membranes.

[174] Sharaf O.Z., Orhan M.F. (2014). An overview of fuel cell technology: Fundamentals and applications. Renew. Sust. Energ. Rev..

[175] Zaman S., Huang L., Douka A.I., Yang H., You B., Xia B.Y. (2021). Oxygen Reduction Electrocatalysts toward Practical Fuel Cells: Progress and Perspectives. Angew. Chem. Int. Ed..

[176] Cullen D.A., Neyerlin K.C., Ahluwalia R.K., Mukundan R., More K.L., Borup R.L., Weber A.Z., Myers D.J., Kusoglu A. (2021). New roads and challenges for fuel cells in heavy-duty transportation. Nat. Energy.

[177] He G.W., Li Z., Zhao J., Wang S.F., Wu H., Guiver M.D., Jiang Z.Y. (2015). Nanostructured Ion-Exchange Membranes for Fuel Cells: Recent Advances and Perspectives. Adv. Mater..

[178] Li W., Liu J., Zhao D.Y. (2016). Mesoporous materials for energy conversion and storage devices. Nat. Rev. Mater..

[179] Ogungbemi E., Ijaodola O., Khatib F.N., Wilberforce T., El Hassan Z., Thompson J., Ramadan M., Olabi A.G. (2019). Fuel cell membranes-Pros and cons. Energy.

[180] Adamski M., Skalski T.J.G., Britton B., Peckham T.J., Metzler L., Holdcroft S. (2017). Highly Stable, Low Gas Crossover, Proton-Conducting Phenylated Polyphenylenes. Angew. Chem. Int. Ed..

[181] Cheng J., He G.H., Zhang F.X. (2015). A mini-review on anion exchange membranes for fuel cell applications: Stability issue and addressing strategies. Int. J. Hydrog. Energy.

[182] Luo T., Abdu S., Wessling M. (2018). Selectivity of ion exchange membranes: A review. J. Membr. Sci..

[183] Wang Y., Ruiz Diaz D.F., Chen K.S., Wang Z., Adroher X.C. (2020). Materials, technological status, and fundamentals of PEM fuel cells—A review. Mater. Today.

[184] Scofield M.E., Liu H.Q., Wong S.S. (2015). A concise guide to sustainable PEMFCs: Recent advances in improving both oxygen reduction catalysts and proton exchange membranes. Chem. Soc. Rev..

[185] Xing L., Shi W., Su H., Xu Q., Das P.K., Mao B., Scott K. (2019). Membrane electrode assemblies for PEM fuel cells: A review of functional graded design and optimization. Energy.

[186] Kim D.J., Jo M.J., Nam S.Y. (2015). A review of polymer–nanocomposite electrolyte membranes for fuel cell application. J. Ind. Eng. Chem..

[187] Tellez-Cruz M.M., Escorihuela J., Solorza-Feria O., Compan V. (2021). Proton Exchange Membrane Fuel Cells (PEMFCs): Advances and Challenges. Polymers.

[188] Wang Y., Chen K.S., Mishler J., Cho S.C., Adroher X.C. (2011). A review of polymer electrolyte membrane fuel cells: Technology, applications, and needs on fundamental research. Appl. Energy.

[189] Zhang J., Liu Y., Lv Z., Zhao T., Li P., Sun Y., Wang J. (2017). Sulfonated Ti3C2Tx to construct proton transfer pathways in polymer electrolyte membrane for enhanced conduction. Solid State Ion..

[190] Yu L., Yue B., Yan L., Zhao H., Zhang J. (2019). Proton conducting composite membranes based on sulfonated polysulfone and polysulfone-g-(phosphonated polystyrene) via controlled atom-transfer radical polymerization for fuel cell applications. Solid State Ion..

[191] Ciftcioglu G.A., Frank C.W. (2021). Effect of Increased Ionic Liquid Uptake via Thermal Annealing on Mechanical Properties of Polyimide-Poly(ethylene glycol) Segmented Block Copolymer Membranes. Molecules.

[192] Watanabe M., Thomas M.L., Zhang S.G., Ueno K., Yasuda T., Dokko K. (2017). Application of Ionic Liquids to Energy Storage and Conversion Materials and Devices. Chem. Rev..

[193] Elwan H.A., Mamlouk M., Scott K. (2021). A review of proton exchange membranes based on protic ionic liquid/polymer blends for polymer electrolyte membrane fuel cells. J. Power Sources.

[194] Schulze M.W., McIntosh L.D., Hillmyer M.A., Lodge T.P. (2014). High-Modulus, High-Conductivity Nanostructured Polymer Electrolyte Membranes via Polymerization-Induced Phase Separation. Nano Lett..

[195] Zhang L.Y., Cui T.T., Cao X., Zhao C.J., Chen Q., Wu L.X., Li H.L. (2017). Inorganic-Macroion-Induced Formation of Bicontinuous Block Co-polymer Nanocomposites with Enhanced Conductivity and Modulus. Angew. Chem. Int. Ed..

[196] Zhai L., Chai S.C., Wang G., Zhang W., He H.B., Li H.L. (2020). Triblock Copolymer/Polyoxometalate Nanocomposite Electrolytes with Inverse Hexagonal Cylindrical Nanostructures. Macromol. Rapid Commun..

[197] Xiao F., Wang Y.C., Wu Z.P., Chen G.Y., Yang F., Zhu S.Q., Siddharth K., Kong Z.J., Lu A.L., Li J.C. (2021). Recent Advances in Electrocatalysts for Proton Exchange Membrane Fuel Cells and Alkaline Membrane Fuel Cells. Adv. Mater..

[198] Guerrero Moreno N., Cisneros Molina M., Gervasio D., Pérez Robles J.F. (2015). Approaches to polymer electrolyte membrane fuel cells (PEMFCs) and their cost. Renew. Sust. Energ. Rev..

[199] Postole G., Auroux A. (2011). The poisoning level of Pt/C catalysts used in PEM fuel cells by the hydrogen feed gas impurities: The bonding strength. Int. J. Hydrog. Energy.

[200] Wang J. (2017). System integration, durability and reliability of fuel cells: Challenges and solutions. Appl. Energy.

[201] Garzon F., Uribe F.A., Rockward T., Urdampilleta I.G., Brosha E.L. (2006). The Impact of Hydrogen Fuel Contaminates on Long-Term PMFC Performance. ECS Trans..

[202] Ramaswamy N., Mukerjee S. (2019). Alkaline Anion-Exchange Membrane Fuel Cells: Challenges in Electrocatalysis and Interfacial Charge Transfer. Chem. Rev..

[203] Varcoe J.R., Atanassov P., Dekel D.R., Herring A.M., Hickner M.A., Kohl P.A., Kucernak A.R., Mustain W.E., Nijmeijer K., Scott K. (2014). Anion-exchange membranes in electrochemical energy systems. Energy Environ. Sci..

[204] Chen N.J., Wang H.H., Kim S.P., Kim H.M., Lee W.H., Hu C., Bae J.Y., Sim E.S., Chung Y.C., Jang J.H. (2021). Poly(fluorenyl aryl piperidinium) membranes and ionomers for anion exchange membrane fuel cells. Nat. Commun..

[205] You W., Noonan K.J.T., Coates G.W. (2020). Alkaline-stable anion exchange membranes: A review of synthetic approaches. Prog. Polym. Sci..

[206] Gao J.F., Wang L., Guo Z., Li B., Wang H., Luo J.C., Huang X.W., Xue H.G. (2020). Flexible, superhydrophobic, and electrically conductive polymer nanofiber composite for multifunctional sensing applications. Chem. Eng. J..

[207] Zhu J.Q., Birgisson B., Kringos N. (2014). Polymer modification of bitumen: Advances and challenges. Eur. Polym. J..

[208] Zhu S., So J.H., Mays R., Desai S., Barnes W.R., Pourdeyhimi B., Dickey M.D. (2013). Ultrastretchable Fibers with Metallic Conductivity Using a Liquid Metal Alloy Core. Adv. Funct. Mater..

[209] Park H.S., Hong C.K. (2021). Anion Exchange Membrane Based on Sulfonated Poly (Styrene-Ethylene-Butylene-Styrene) Copolymers. Polymers.

[210] Zhu M., Su Y.X., Wu Y.B., Zhang M., Wang Y.G., Chen Q., Li N.W. (2017). Synthesis and properties of quaternized polyolefins with bulky poly(4-phenyl-1-butene) moieties as anion exchange membranes. J. Membr. Sci..

[211] Zhang X.J., Chu X.M., Zhang M., Zhu M., Huang Y.D., Wang Y.G., Liu L., Li N.W. (2019). Molecularly designed, solvent processable tetraalkylammonium-functionalized fluoropolyolefin for durable anion exchange membrane fuel cells. J. Membr. Sci..

[212] Pan Y., Jiang K., Sun X.R., Ma S.Y., So Y.M., Ma H.W., Yan X.M., Zhang N., He G.H. (2021). Facilitating ionic conduction for anion exchange membrane via employing star-shaped block copolymer. J. Membr. Sci..

[213] Tarascon J.M., Armand M. (2001). Issues and challenges facing rechargeable lithium batteries. Nature.

[214] Sun Y.M., Liu N.A., Cui Y. (2016). Promises and challenges of nanomaterials for lithium-based rechargeable batteries. Nat. Energy.

[215] Ye Y.S., Chou L.Y., Liu Y.Y., Wang H.S., Lee H.K., Huang W.X., Wan J.Y., Liu K., Zhou G.M., Yang Y.F. (2020). Ultralight and fire-extinguishing current collectors for high-energy and high-safety lithium-ion batteries. Nat. Energy.

[216] Liu K., Liu Y., Lin D., Pei A., Cui Y. (2018). Materials for lithium-ion battery safety. Sci. Adv..

[217] Lee H., Yanilmaz M., Toprakci O., Fu K., Zhang X.W. (2014). A review of recent developments in membrane separators for rechargeable lithium-ion batteries. Energy Environ. Sci..

[218] Zhang L.P., Li X.L., Yang M.R., Chen W.H. (2021). High-safety separators for lithium-ion batteries and sodium-ion batteries: Advances and perspective. Energy Storage Mater..

[219] Zhang Y., Yuan J.J., Song Y.Z., Yin X., Sun C.C., Zhu L.P., Zhu B.K. (2018). Tannic acid/polyethyleneimine-decorated polypropylene separators for Li-Ion batteries and the role of the interfaces between separator and electrolyte. Electrochim. Acta.

[220] Wang X., Peng L.Q., Hua H.M., Liu Y.Z., Zhang P., Zhao J.B. (2020). Magnesium Borate Fiber Coating Separators with High Lithium-Ion Transference Number for Lithium-Ion Batteries. Chemelectrochem.

[221] Zahn R., Lagadec M.F., Hess M., Wood V. (2016). Improving Ionic Conductivity and Lithium-Ion Transference Number in Lithium-Ion Battery Separators. ACS Appl. Mater. Interfaces.

[222] Ren D.S., Feng X.N., Liu L.S., Hsu H.J., Lu L.G., Wang L., He X.M., Ouyang M.G. (2021). Investigating the relationship between internal short circuit and thermal runaway of lithium-ion batteries under thermal abuse condition. Energy Storage Mater..

[223] Zheng Q.F., Yamada Y., Shang R., Ko S., Lee Y.Y., Kim K., Nakamura E., Yamada A. (2020). A cyclic phosphate-based battery electrolyte for high voltage and safe operation. Nat. Energy.

[224] Zhang X.Q., Chen X., Cheng X.B., Li B.Q., Shen X., Yan C., Huang J.Q., Zhang Q. (2018). Highly Stable Lithium Metal Batteries Enabled by Regulating the Solvation of Lithium Ions in Nonaqueous Electrolytes. Angew. Chem. Int. Ed..

[225] Yang H., Shi X.S., Chu S.Y., Shao Z.P., Wang Y. (2021). Design of Block-Copolymer Nanoporous Membranes for Robust and Safer Lithium-Ion Battery Separators. Adv. Sci..

[226] Wang Y., Zhong W.H. (2015). Development of Electrolytes towards Achieving Safe and High-Performance Energy-Storage Devices: A Review. Chemelectrochem.

[227] Xiao Y.R., Turcheniuk K., Narla A., Song A.Y., Ren X.L., Magasinski A., Jain A., Huang S., Lee H., Yushin G. (2021). Electrolyte melt infiltration for scalable manufacturing of inorganic all-solid-state lithium-ion batteries. Nat. Mater..

[228] Schnell J., Gunther T., Knoche T., Vieider C., Kohler L., Just A., Keller M., Passerini S., Reinhart G. (2018). All-solid-state lithium-ion and lithium metal batteries-paving the way to large-scale production. J. Power Sources.

[229] Wang Q.S., Jiang L.H., Yu Y., Sun J.H. (2019). Progress of enhancing the safety of lithium ion battery from the electrolyte aspect. Nano Energy.

[230] Manthiram A., Yu X.W., Wang S.F. (2017). Lithium battery chemistries enabled by solid-state electrolytes. Nat. Rev. Mater..

[231] Lin Z.Y., Guo X.W., Yang Y.B., Tang M.X., Wei Q., Yu H.J. (2021). Block copolymer electrolyte with adjustable functional units for solid polymer lithium metal battery. J. Energy Chem..

[232] He Y.B., Liu N., Kohl P.A. (2021). Difunctional block copolymer with ion solvating and crosslinking sites as solid polymer electrolyte for lithium batteries. J. Power Sources.

[233] Liu D., Yang W.L., Liu Y., Yang S.C., Shen Z.H., Fan X.H., Yang H., Zhou Q.F. (2022). Enhancing ionic conductivity in tablet-bottlebrush block copolymer electrolytes with well-aligned nanostructures via solvent vapor annealing. J. Mater. Chem. C.

